# Association between altered tryptophan metabolism, plasma aryl hydrocarbon receptor agonists, and inflammatory Chagas disease

**DOI:** 10.3389/fimmu.2023.1267641

**Published:** 2024-01-12

**Authors:** Laura Fernanda Ambrosio, Ximena Volpini, Juan Nahuel Quiroz, María Belén Brugo, Carolina Paola Knubel, Melisa Rocío Herrera, Laura Fozzatti, Julián Avila Pacheco, Clary B. Clish, Maisa C. Takenaka, Juan Beloscar, Martín Gustavo Theumer, Francisco Javier Quintana, Ana Rosa Perez, Claudia Cristina Motrán

**Affiliations:** ^1^Departamento de Bioquímica Clínica, Facultad de Ciencias Químicas, Universidad Nacional de Córdoba, Córdoba, Argentina; ^2^Centro de Investigaciones en Bioquímica Clínica e Inmunología (CIBICI), Consejo Nacional de Investigaciones Científicas y Técnicas (CONICET), Córdoba, Argentina; ^3^Broad Institute of Massachusetts Institute of Technology (MIT) and Harvard, Cambridge, MA, United States; ^4^Ann Romney Center for Neurologic Diseases, Brigham and Women’s Hospital, Harvard Medical School, Boston, MA, United States; ^5^Servicio de Cardiología, Departamento de Chagas, Hospital Provincial del Centenario y Cátedra de Cardiología, Facultad de Ciencias Médicas, Universidad Nacional de Rosario, Rosario, Argentina; ^6^Instituto de Inmunología Clínica y Experimental de Rosario-CONICET-Universidad Nacional de Rosario (IDICER-CONICET-UNR), Rosario, Argentina; ^7^Centro de Investigación y Producción de Reactivos Biológicos (CIPReB), Facultad de Ciencias Médicas, Universidad Nacional de Rosario, Rosario, Argentina

**Keywords:** Chagas, AhR, tryptophan, IDO, *T. cruzi*, metabolomic

## Abstract

**Introduction:**

Chagas disease causes a cardiac illness characterized by immunoinflammatory reactions leading to myocardial fibrosis and remodeling. The development of Chronic Chagas Cardiomyopathy (CCC) in some patients while others remain asymptomatic is not fully understood, but dysregulated inflammatory responses are implicated. The Aryl hydrocarbon receptor (AhR) plays a crucial role in regulating inflammation. Certain tryptophan (Trp) metabolites have been identified as AhR ligands with regulatory functions.

**Methods, results, and discussion:**

We investigated AhR expression, agonist response, ligand production, and AhR-dependent responses, such as IDO activation and regulatory T (Treg) cells induction, in two T. cruzi-infected mouse strains (B6 and Balb/c) showing different polymorphisms in AhR. Furthermore, we assessed the metabolic profile of Trp catabolites and AhR agonistic activity levels in plasma samples from patients with chronic Chagas disease (CCD) and healthy donors (HD) using a luciferase reporter assay and liquid chromatography-mass spectrophotometry (LC-MS) analysis. T. cruzi-infected B6 mice showed impaired AhR-dependent responses compared to Balb/c mice, including reduced IDO activity, kynurenine levels, Treg cell induction, CYP1A1 up-regulation, and AhR expression following agonist activation. Additionally, B6 mice exhibited no detectable AhR agonist activity in plasma and displayed lower CYP1A1 up-regulation and AhR expression upon agonist activation. Similarly, CCC patients had decreased AhR agonistic activity in plasma compared to HD patients and exhibited dysregulation in Trp metabolic pathways, resulting in altered plasma metabolite profiles. Notably, patients with severe CCC specifically showed increased N-acetylserotonin levels in their plasma. The methods and findings presented here contribute to a better understanding of CCC development mechanisms and may identify potential specific biomarkers for T. cruzi infection and the severity of associated heart disease. These insights could be valuable in designing new therapeutic strategies. Ultimately, this research aims to establish the AhR agonistic activity and Trp metabolic profile in plasma as an innovative, non-invasive predictor of prognosis for chronic Chagas disease.

## Introduction

Chagas disease, caused by the protozoan parasite *Trypanosoma cruzi*, is a significant public health issue in endemic regions spanning from Mexico to southern Argentina. Furthermore, Chagas disease is responsible for approximately 12,000 deaths annually, with most of them attributed to the development of chronic Chagas cardiomyopathy (CCC). CCC affects around 30% of individuals infected with the disease and typically manifests several decades after the acute infection (1, 2). Approximately 10% of chronic Chagas disease (CCD) patients, equivalent to nearly one-third of CCC patients, develop a severe and potentially fatal form of dilated cardiomyopathy characterized by ventricular dysfunction, heart failure, and arrhythmias. The remaining individuals either remain asymptomatic (IND) or develop digestive disorders (5-10%) without cardiac involvement. While the precise mechanisms governing the divergent progression to CCC remain incompletely understood, it is widely accepted that CCC patients exhibit an imbalance in the production of inflammatory and anti-inflammatory cytokines, as well as a heightened pro-inflammatory response compared to IND patients. CCC patients exhibit elevated levels of circulating IL-6 and TNF, increased numbers of CD4+ and CD8+ T cells producing IFN-γ, and decreased numbers of IL-10-producing CD4+ T cells and CD4+CD25+ Foxp3+ regulatory T (Treg) cells in their peripheral blood compared to IND patients (3–9). Moreover, heart tissue samples from CCC patients reveal an inflammatory infiltrate rich in Th1 cells that predominantly secrete IFN-γ and TNF (4, 10–14). These findings suggest that immunoregulatory mechanisms may play a role in modulating the intensity of inflammation to prevent the development of CCC.

During experimental *T. cruzi* infection, host resistance relies on the rapid induction of a Th1 inflammatory response. However, this response must be carefully balanced to prevent inflammation-mediated pathology and ensure survival. Both C57BL/6 (B6) and Balb/c mice infected with the Tulahuen strain of *T. cruzi* exhibit acute disease characterized by splenomegaly. However, B6 mice demonstrate a progressive and ultimately fatal disease, while Balb/c mice experience a partial recovery (15). In line with the observations in patients with CCC, B6 mice exhibit significant challenges in controlling the inflammatory response. In these mice, the liver becomes the primary target of pathological inflammatory damage, and infected mice experience premature death due to liver failure (15). Interestingly, the mortality in B6 mice does not appear to be caused by an exacerbated infection, as the parasite load is actually lower compared to that observed in Balb/c mice (15). One significant distinction between these two mouse strains lies in their systemic cytokine levels after the infection. B6 mice have higher levels of TNF and lower levels of IL-10 compared to Balb/c mice (15). Another important difference is the inability of B6 mice to expand the population of Treg cells in proportion to the extensive expansion of the T cell compartment. This leads to an increased ratio of T effector cells to Treg cells (16, 17). Collectively, these findings suggest that the fatal outcome observed in B6 mice may be attributed to an imbalanced Th1 response, which is likely due to inadequate induction of regulatory mechanisms.

Indoleamine 2,3-dioxygenase (IDO) is an intracellular enzyme that plays a role in the degradation of the essential amino acid tryptophan (Trp) in the kynurenine (KYN) pathway, resulting in the production of various biologically active catabolites known as kynurenines (18). We have reported that IDO activity is up-regulated following *T. cruzi* infection in mice. Furthermore, we have observed that blocking IDO activity impairs resistance to the infection, and that *T. cruzi* amastigotes and trypomastigotes are susceptible to 3-hydroxykynurenine (3-HK), a metabolite of the KYN pathway (19–21). Additionally, treatment with 3-HK in *T. cruzi*-infected Balb/c mice significantly reduces the incidence and severity of inflammatory pathology. This treatment also modulates the immune response by impairing Th1 and Th2 specific responses, while promoting the induction of TGF-β-secreting cells and Treg cells (19, 20).

It has been reported that the induction of IDO depends on the expression of aryl hydrocarbon receptor (AhR) (22). Furthermore, the production of KYN can activate AhR, and there exists a positive feedback loop where increased IDO expression leads to Trp degradation and KYN production (23). AhR is a ligand-activated transcription factor that plays crucial roles in various biological processes, including development, detoxification, and immune response (24). In the absence of ligands, AhR is located in the cytoplasm complexed with chaperones. Upon ligand binding, the receptor translocates to the nucleus and forms a complex with its partner protein, aryl hydrocarbon receptor nuclear translocator (ARNT), to regulate the transcription of target genes. These include Phase I drug metabolizing enzymes like CYP1A1, which contain responsive elements (AhREs) in their promoter regions (24, 25). In addition to its role in genes with AhREs, AhR also participates in the transcriptional regulation of genes that do not possess AhREs, such as NF-κB and STAT proteins, highlighting its function as an immunomodulator (26–28). Overall, AhR activation plays a crucial role in modulating both innate and adaptive immune responses by interacting with multiple AhREs found in immune-related genes. It acts as a key regulator that maintains the balance between regulatory T cells (Tregs, including Tr1 and CD4+CD25+Foxp3+ Treg cells) and Th17 cells (27, 29, 30).

Exogenous xenobiotics like TCDD and endogenous ligands such as FICZ and ITE have been shown to activate AhR in immune cells (31). Additionally, several physiological AhR ligands derived from Trp have been identified, including KYN and 3-HK, which activate AhR in lymphoid tissues and promote Treg cells development (32–34). Notably, our recent findings suggest that AhR activation is involved in controlling the exacerbated Th1 cell response during *T. cruzi* infection (35). One of the key questions regarding AhR activation is how various ligands induce different immunological changes (36). Multiple factors contribute to the effects observed upon AhR activation, including the characteristics of the ligand, the specific cell type expressing AhR, and the presence of coactivators (22, 37, 38). Genetic polymorphisms in the AhR gene can also influence the responsiveness to AhR ligands (39). B6 and Balb/c mice, for example, express AhRb-1 and AhRb-2 alleles, respectively, and both strains have been classified as high responders based on TCDD-induced CYP1A1 expression (39). However, when exposed to weaker AhR agonists than TCDD, Balb/c mice exhibit higher responses compared to B6 mice (39, 40). Therefore, the differential outcomes of *T. cruzi* infection may be linked to the presence of different AhR alleles, which exhibit varying strengths and extents of activation. This, in turn, could result in differential production of AhR ligands and subsequently modulate the regulatory response.

Our hypothesis proposes that during *T. cruzi* infection, differential AhR expression and/or activation play a crucial role in regulating the production of endogenous AhR ligands. This, in turn, influences the induction of regulatory mechanisms that can effectively control the pro-inflammatory response and mitigate the development of pathology. Ultimately, these factors contribute to shaping the overall outcome of the infection.

## Materials and methods

### Statement of ethics

This study was designed and performed according to the registration and had been approved by the Ethics Committee of the Facultad de Medicina de la Universidad Nacional de Rosario, Santa Fe, Argentina (RES N°: 666/2015). Gender-matched mice that were aged between 6 to 8 weeks were used for the experiment. All animal experiments were approved by and conducted in accordance with the guidelines of the Animal Care and Use Committee of the Facultad de Ciencias Químicas, Universidad Nacional de Córdoba (Approval Number HCD 743/18).

### Mice and parasites

C57BL/6 (B6) and Balb/c mice were originally obtained from the Facultad de Veterinaria de la Universidad Nacional de La Plata (La Plata, Argentina) and were housed in the Animal Facility of the Facultad de Ciencias Químicas, Universidad Nacional de Córdoba (OLAW Assurance number A5802-01). Female and male B6 and Balb/c mice (6-8 weeks old) were intraperitoneally injected with 50,000 bloodstream trypomastigotes (Tps) of *T. cruzi-*Tulahuen strain as described previously (41). For each experiment, all animals were infected on the same day and subsequently euthanized at different days post-infection (dpi). *T. cruzi* was maintained by weekly intraperitoneal inoculation in Balb/c mice. For *in vitro* infections, cell culture-derived Tps were obtained from the initial burst of infected Vero cell monolayers.

### Macrophages and cell lines

Bone marrow-derived macrophages (BMDM) and peritoneal macrophages (PM) were obtained as previously described by Hsu et al. and Motrán et al., respectively (42, 43). HEK293 and 4T1 cell lines were obtained from the American Type Culture Collection (Manassas, VA, USA). Mice splenocytes from non-infected (NI) or infected (INF) mice were obtained as we described previously (35).

### Cell culture reagents

Macrophages were cultured in RPMI 1640 Medium supplemented with 2 mM GlutaMAX, 10% heat-inactivated fetal bovine serum, and 50 μg/mL Gentamicin. 4T1 (ATCC Cat# CRL-2539, RRID:CVCL_0125) and HEK-293 (ATCC Cat# CRL-1573, RRID:CVCL_0045) cell lines were cultured with Dulbecco’s modified eagle medium (DMEM) supplemented with 50 μg/mL gentamicin. All culture media and PBS were purchased from Gibco, Thermo Fisher Scientific; bovine serum albumin and DMSO were obtained from Sigma-Aldrich.

### Flow cytometry

Dissected spleen from mice was harvested in PBS using a 70µm cell-strainer (Merck) and collected in tubes. The whole suspension was centrifuged for 5 minutes, at 4°C and 200 xg. The cell pellet was subjected to RBC lysis buffer (eBioscience) for 5 minutes and washed by centrifugation with PBS. After that, spleen cells were counted and 2x10^6^ cells were incubated with fluorochrome-labeled antibodies for superficial and intracellular markers as described previously (35). Different combinations of the following antibodies were used: CD4-FITC, CD25-PECy7, Foxp3-PE (eBioscience). Viability was inquired with APC-Cy7 Zombie NIRTM kit (Biolegend). Samples were acquired in a FACSCanto II (BD Biosciences), and the data were analyzed using FlowJO V10 software. After excluding doublets and death cells, Treg was identified as CD3+CD4+CD25+Foxp3+ and T effector cells as CD3+CD4+CD25-Foxp3-. The absolute number of each population was calculated by referring to the total number of spleen cells. The gating strategies to identify Treg and T effector cells are shown in **Supplementary Figure 1**.

### In vitro infection and AhR activation treatments

BMDM were cultured for 24 h in complete RPMI medium alone or with TCDD (160 nM), ITE (100 nM), *T. cruzi* total lysate (10 ug/ml), or total lysate plus CH223191 (3 µM). RNA extraction and qPCR analysis for CYP1A1 and AhR were performed.

For RNA-seq, BMDM were infected with *T. cruzi* (1:3 ratio, BMDM:Tps) for 24 h, followed by washing with PBS to remove non-internalized Tps as described previously (19, 44). Non-infected BMDM served as controls. RNA-seq was conducted using the High-Throughput 3’ Digital Gene Expression (HT-DGE) method at the Broad Institute. Analysis of the RNA-seq data was carried out using Ingenuity Pathway Analysis (Qiagen) to identify differential expression of AhR pathway-related genes during infection.

### Patients cohorts’ formation

This study included healthy donors (HD) and chronic Chagas disease (CCD) patients aged 21-68 years, examined at the Cardiology Unit, Chagas Department of the Century Hospital, Faculty of Medical Science of the National University of Rosario. The infection was confirmed by two positive tests (ELISA, hemagglutination, and immunofluorescence). Seropositive patients underwent an electrocardiogram (ECG) and chest X-ray (CXR) to classify them as indeterminate (IND) or with Chronic Chagas Cardiomyopathy (CCC), with CCC further classified as Mild or Severe based on the results. None of the individuals with Chagas disease included in this study had received treatment with either Benznidazole or Nifurtimox. Exclusion criteria included immunological disease, hormonal and immunomodulatory treatments, and known genetic alterations. Patients were not receiving specific treatment or presenting concomitant pathologies.

### Mice and human plasma collection

Blood was obtained by cardiac puncture of mice under deep anesthesia. For plasma collection, the blood samples were centrifuged at 200 xg for 5 min. Human plasma was obtained by venipuncture. Blood samples were placed in a 15 ml conical tube containing heparin and then were centrifuged for 5 min at 200 xg for plasma collection.

### Western blot

Spleen cell samples from mice were separated by gel electrophoresis using a 10% acrylamide gel. The separated proteins were then transferred onto a nitrocellulose membrane. To block non-specific binding, the membrane was treated with 5% skim milk. Overnight incubation with anti-AhR (Enzo Life Sciences Cat# BML-SA550, RRID:AB_2223953) or anti-β-actin (Abcam Cat# ab25894, RRID:AB_448882) antibodies was carried out. After washing, the membranes were exposed to the corresponding IRD Fluor 800-labeled IgG (LI-COR Biosciences Cat# 925-32211, RRID:AB_2651127) or IRD Fluor 680-labeled IgG secondary antibody (LI-COR Biosciences Cat# 926-68050, RRID:AB_2783642) for 1 hour at room temperature. The membranes were then scanned using the Odyssey infrared imaging system (Odyssey CLx, RRID:SCR_014579), and the obtained images were analyzed using Image Studio 3.1 software for densitometric analysis.

### Quantitative real-time PCR

RNA was extracted from BMDM and splenocytes using Trizol reagent (Invitrogen, Thermo Fisher Scientific) following the manufacturer’s instructions. cDNA was generated by reverse transcription, and specific genes were quantified by quantitative real-time PCR (qPCR) using SYBR Green (Thermo Fisher Scientific) with the following primers: *Ahr* 5′ GAA GGA GAG TTC TTG TTA CAG GCG 3′ (forward), and 5′ GGA GGA AGC ATA GAA GAC CAA GG 3′ (reverse); *Cyp1a1* 5′ CAT TCC TGT CCT CCG TTA CCT G 3′ (forward), and 5′ CTG TCT GTG ATG TCC CGG ATG 3′ (reverse); and *Actb* 5′ CGC CAC CAG TTC GCC ATG GA 3′ (forward), and 5′ TAC AGC CCG GGG AGC ATC GT 3′ (reverse). The relative expression of *Ahr* and *Cyp1a1* was normalized to *Actb* (β-actin) by using delta-delta-CT method, and expressed as mRNA relative levels. The qPCR amplification was performed in a StepOnePlus™ thermocycler (Thermo Fisher Scientific), under the following amplification conditions: 95°C for 10 min, 45 cycles x (95°C for 15 s and 60°C for 1 min), and 72°C for 5 min.

### Determination of AhR agonistic activity

HEK-293 or 4T1 cells were seeded at a density of 15,000 cells per well in flat-bottom 96-well plates. The cells were transfected with pGud-Luc (Firefly luciferase under the control of an AHR-responsive promoter element) and pTK-Renilla (Renilla luciferase under the control of a constitutively active thymidine kinase promoter; Promega, Madison, WI) using Fugene Transfection Reagent (Promega), following the manufacturer’s instructions. After 24 hours, the transfected cells were treated with DMEM supplemented with 10% human or mouse plasma in triplicates. Luciferase activity was measured 24 hours later using the Dual-Luciferase Reporter System (Promega). The Firefly luciferase activity was normalized to Renilla luciferase activity and expressed as a percentage relative to the control levels, which were set as 100% (45).

### HPLC analysis of KYN

Serum and potassium phosphate buffer (0.05M, pH 6.0) containing the internal calibrator 3-nitro-L-tyrosine (100μM) were mixed in equal amounts, as previously described (Knubel, 2010) (19). Proteins were precipitated by adding 2 M trichloroacetic acid, followed by vortex-mixing and centrifugation at 13,000 xg for 10 minutes. The supernatant was collected and subjected to analysis by HPLC using a Hewlett Packard HP1100 series system (Hewlett Packard, Palo Alto, CA, USA).

### IDO activity assay

Spleen cells from mice were suspended in PBS and sonicated for 30 seconds at 4°C. The cell homogenate was centrifuged at 800 xg for 10 minutes at 4°C, and the resulting supernatant was collected. This supernatant was further centrifuged at 15,000 xg for 15 minutes at 4°C. The resulting supernatant was collected and utilized for the colorimetric assay, as described by Kudo and Boyd (46).

### Plasma metabolite analysis

Plasma metabolites were measured using targeted a liquid chromatography tandem mass spectrometry (LC-MS) configured on a 1290 Infinity II U-HPLC coupled to an Agilent 6495 Triple Quadrupole mass spectrometer (Agilent Tech. Santa Clara, CA). Metabolites were extracted from plasma (10 µL) using 90 µL of acetonitrile/methanol/formic acid (74.9:24.9:0.2 v/v/v) containing stable isotope-labeled internal standards (valine-d8, Sigma-Aldrich; St. Louis, MO; and phenylalanine-d8, Cambridge Isotope Laboratories, Andover, MA). The samples were centrifuged (10 min, 9,000 x g, 4°C), and the supernatants were injected directly onto a 150 x 2 mm, 3 µm Atlantis HILIC column (Waters; Milford, MA). The column was eluted isocratically at a flow rate of 250 µL/min with 5% mobile phase A (10 mM ammonium formate and 0.1% formic acid in water) for 0.5 minute followed by a linear gradient to 40% mobile phase B (acetonitrile with 0.1% formic acid) over 10 minutes. Multiple reaction monitoring MS parameters were determined using authentic reference standards for every metabolite. Peak abundances were manually integrated using the MassHunter software provided by the LC-MS manufacturer and visually reviewed to ensure quality.

### Statistical analysis

All the statistical analysis was performed using Prism 7 software (GraphPad). Data distribution was analyzed with the Shapiro-Wilk test. Normally distributed data were presented as mean± SD, and the differences between the mean values were assessed using Student’s t-test or One Way ANOVA followed by Tukey’s post-test. Non-normally distributed data were analyzed using the Man-Whitney U test (non-parametric) and presented as the median and interquartile range (IQR). Results were considered significantly different when *p* < 0.05. PCA analysis was performed by using the FactoMiner package (v2.8). A correlation matrix for Trp-related metabolites was created with corrplot package (v0.92). Correlation plots between Kyn and Trp showing Spearman’s rank coefficient (ρ) and CI 95% were elaborated using ggstatsplot package (v0.12.0). The mentioned packages were run in Rstudio (v2023.06.0).

## Results

### Impaired up-regulation of IDO activity and Treg in B6 mice

Trp, an essential amino acid obtained from the diet, can be metabolized by both commensal bacteria enzymes and the host’s Trp-degrading enzymes, such as IDO and L-tryptophan 2,3-dioxygenase (TDO), leading to the production of various AhR ligands (24, 47, 48). Our research has demonstrated that *T. cruzi* experimental infection up-regulates the activity of IDO, resulting in the generation of several biologically active catabolites (19), some of them identified as AhR ligands. The expression of AhR is crucial for the induction of IDO in dendritic cells (DC) (22, 49), and it has been suggested that IDO activity and the production of kynurenines play a role in controlling excessive inflammatory responses (50–53). Furthermore, the IDO gene promoter contains DNA sequences that respond to pro-inflammatory mediators, highlighting the strong association between inflammation and IDO expression (54).

To investigate the relationship between the inflammatory status of *T. cruzi*-infected Balb/c and B6 mice and the induction of IDO activity and Treg cells, we assessed IDO activity in spleen mononuclear cells (SMC), measured KYN levels in serum, and analyzed the population of Treg cells in the spleen at various time points post-infection (p.i.).

**Figure 1:**
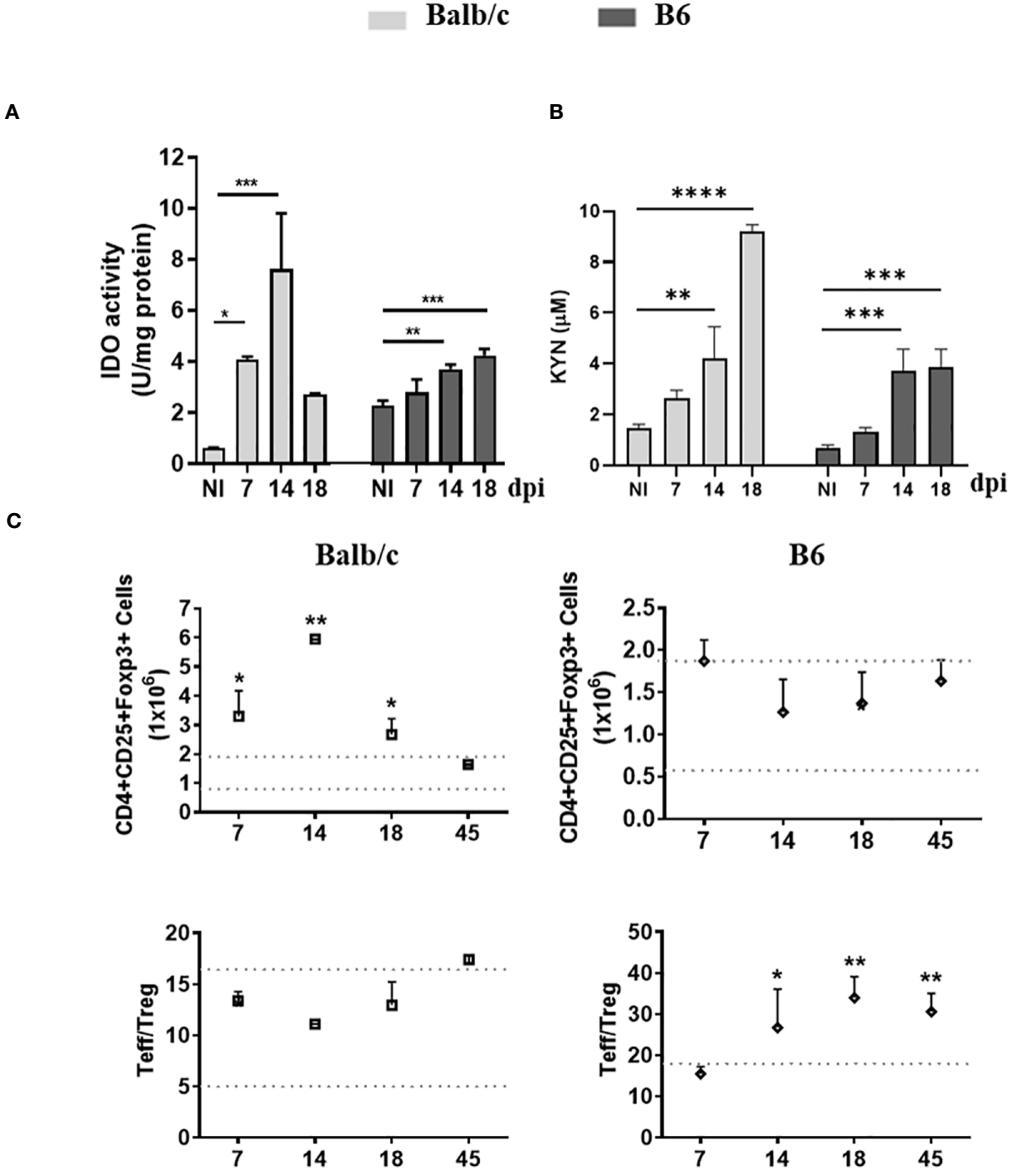
AhR-dependent features, up-regulation of IDO activity and Treg promotion, are Impaired in B6 mice during T. cruzi infection. **(A)** IDO activity was determined in spleen extracts of non-infected (NI) or T. cruzi-infected Balb/c and B6 mice at different time points p.i. The bars represent the mean ± SD activity (U/mg protein) with three mice per time point. **(B)** KYN concentration in plasma was measured using HPLC. The bars represent the mean ± SD with three mice per time point. The statistical significance was calculated by comparing each time point to the NI of the respective group and is denoted by (*)p<0.05, (**)p<0.001, and (****)p<0.0001. **(C)** The absolute number of splenic Treg cells (CD4+ CD25+ Foxp3+) is shown in the upper panels, while the ratio of splenic effector T cells (CD4+ CD25-) to Treg cells is shown in the lower panels. Statistical calculations were performed using one-way ANOVA followed by Dunnett’s test. The significance levels are denoted by (*)p<0.05 and (**)p<0.001. Each data point represents the mean ± SD with three mice per time point. In **(A-C)** the results shown are representative of three independent experiments.

Interestingly, despite exhibiting a more inflammatory profile compared to Balb/c mice, B6 mice demonstrated a lower induction of IDO activity with a delayed kinetic (**Figure 1A**). Consistent with this reduced IDO activity, lower levels of KYN were observed in the plasma of B6 mice compared to Balb/c mice (**Figure 1B**).

Furthermore, unlike B6 mice, Balb/c mice exhibited an expansion of the Treg population alongside the substantial expansion of the T cell compartment, leading to a T effector/Treg cell ratio like that observed in non-infected mice (NI) (**Figure 1C**). These findings suggest that the IDO-dependent negative feedback loop, which plays a role in regulating the inflammatory response, is not adequately induced in B6 mice.

### Differential modulation of AhR expression on spleen cells from Balb/c and B6 mice during T. cruzi infection

**Figure 2:**
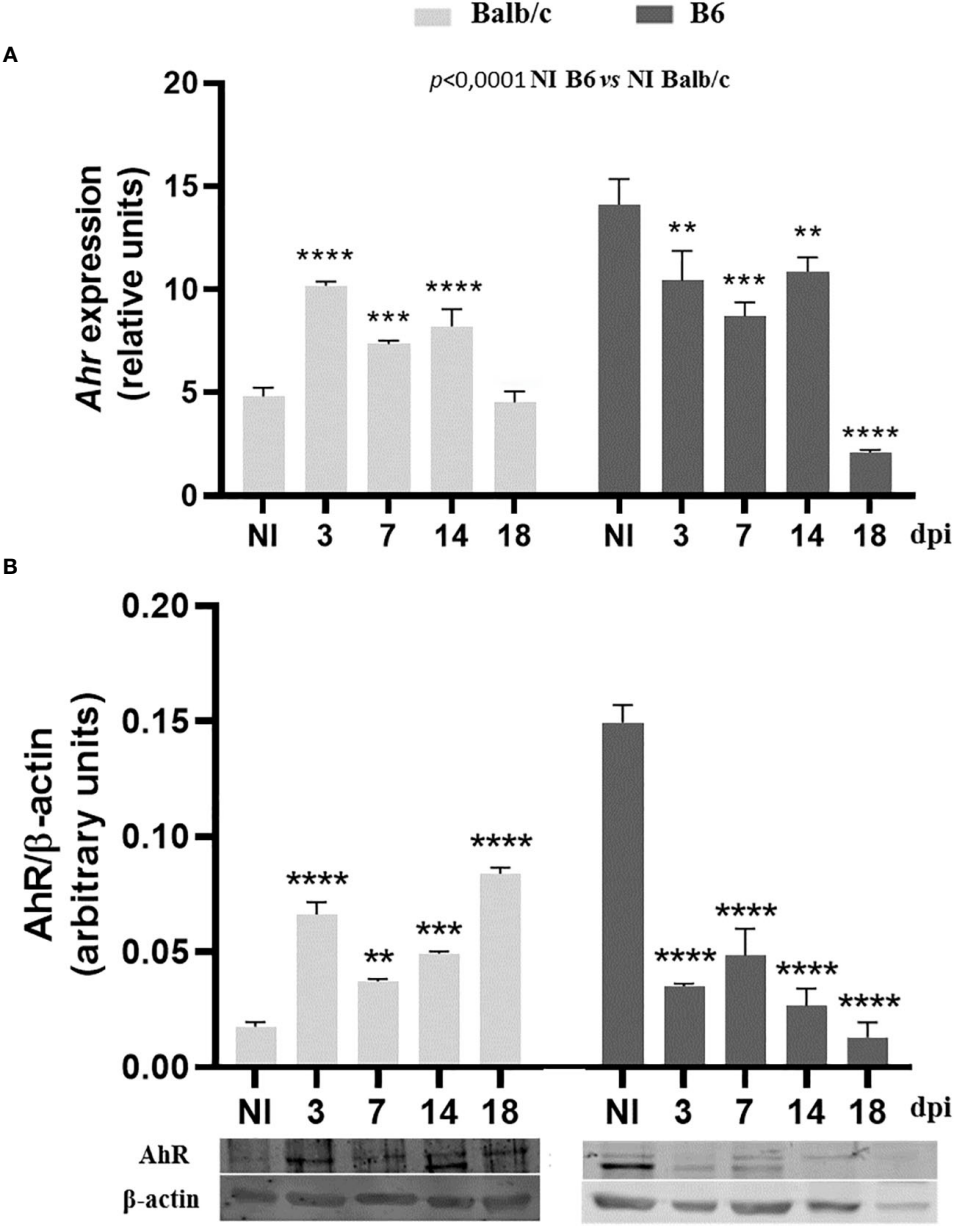
Differential modulation of AhR expression on spleen cells from Balb/c and B6 mice during T. cruzi infection. Splenocytes from non-infected (NI) or T. cruzi-infected Balb/c and B6 mice were collected at various time points p.i. to evaluate AhR expression. **(A)** AhR mRNA expression was determined using qPCR. The data are presented as the mean ± SD of AhR relative expression normalized to Actb expression (β-actin) from 3 mice per time point. **(B)** AhR protein expression was assessed by Western blot at different times p.i. Each bar represents the mean ± SD of protein relative expression levels, quantified by scanning the intensity of band areas in the homogenates and normalized to β-actin (n = 3 mice per time point). P-values were calculated using one-way ANOVA followed by Dunnett’s test, comparing the NI of each group vs. infected samples at different times p.i.; (*)p<0.01; (**)p<0.001; (***)p<0.001. The results presented are representative of those obtained in two independent experiments.

To investigate the expression of AhR and its modulation during *T. cruzi* infection, we assessed AhR mRNA and protein levels in SMC at different time points p.i. using quantitative qPCR and Western blot analysis. We observed that uninfected B6 mice exhibited higher levels of AhR expression compared to Balb/c mice (*p* ≤ 0.0001, **Figure 2A**). Interestingly, *T. cruzi* infection led to a significant up-regulation of AhR expression in splenocytes from Balb/c mice, while a down-regulation of AhR expression was observed in splenocytes from B6 mice (**Figures 2A, B**). These findings highlight the differential modulation of AhR expression in response to *T. cruzi* infection in the two mouse strains.

### Macrophages of Balb/c and B6 mice show different responsiveness to AhR ligands

In the B6 model of *T. cruzi* experimental infection, which is characterized by a higher inflammatory response compared to the Balb/c model, we observed significant differences in the expression of AhR, IDO activity, KYN levels, and Treg cell levels.

Given that AhR expression and AhR polymorphisms can both influence the response to AhR ligands (39), we hypothesized that the observed variations in IDO activity and Treg cell levels between Balb/c and B6 mice after *T. cruzi* infection could be attributed at least in part to the differences in AhR polymorphisms between these strains. Specifically, Balb/c mice carry the AhRb-2 variant, while B6 mice carry the AhRb-1 variant.

**Figure 3:**
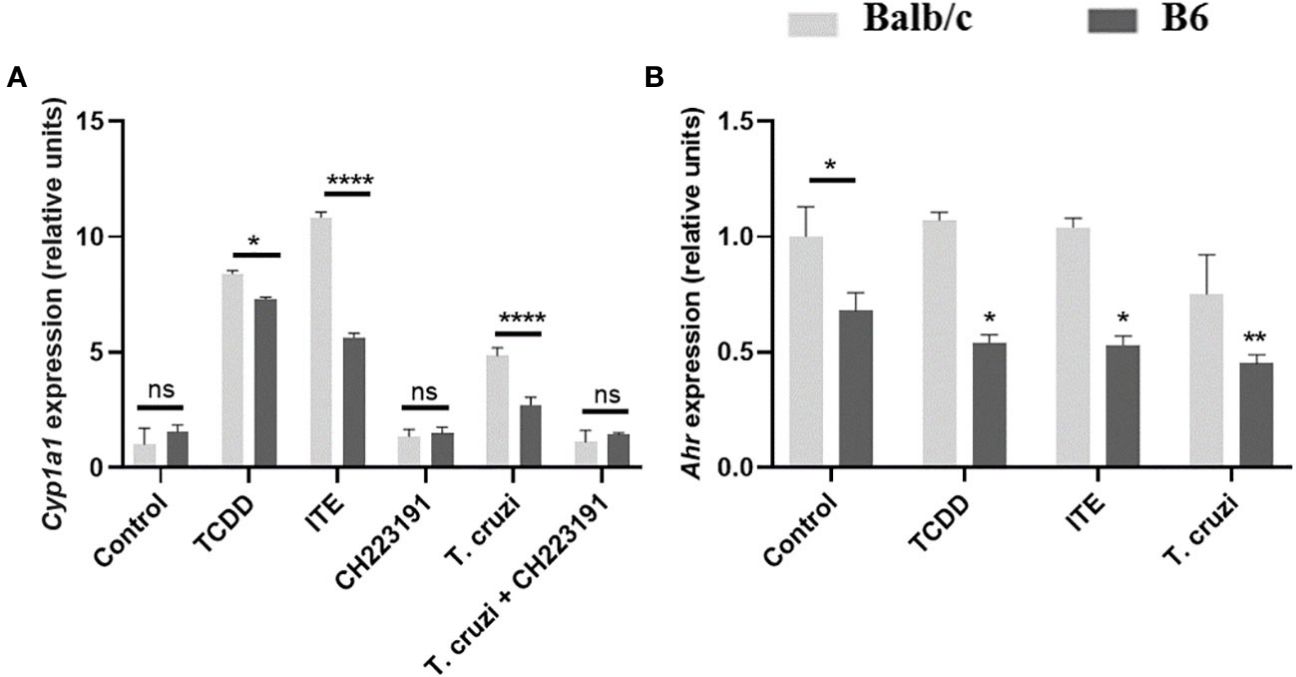
B6 and Balb/c mice exhibit differential responsiveness to AhR ligands. BMDM from Balb/c and B6 mice were cultured for 24 hours with the AhR agonists TCDD (160 nM), ITE (100 nM), and T. cruzi lysate (10µg/ml) +/- CH223191 (3 µM). **(A)** Cyp1a1 expression was assessed by qPCR. **(B)** Ahr expression was determined by qPCR. Each bar represents the mean ± SD of Cyp1a1/Ahr gene expression levels normalized to Actb expression (β-actin). Values are the means of triplicate measurements for experiment. P values were calculated using Student’s t-test. Simple (*) indicates comparations of each condition vs the control within the respective group (Balb/c or B6), while lines represent comparations between the specified bars. (*)p ≤ 0.05, (**)p ≤ 0.01, and (***)p ≤ 0.0001. ns, non-significant. The results presented are representative of those obtained in three independent experiments.

To test this polymorphism hypothesis, we treated bone marrow-derived macrophages (BMDM) from Balb/c and B6 mice with AhR ligands TCDD and ITE, as well as *T. cruzi* lysate, for 24 hours. We then measured the expression of *Ahr* and *Cyp1a1*, as indicators of the AhR response to its ligands, in each strain by qPCR. Upon stimulation with TCDD, Balb/c BMDM showed approximately an 8-fold increase in *Cyp1a1* expression, while B6 BMDM showed approximately a 5-fold increase (**Figure 3A**). Similar results were observed following stimulation with ITE or *T. cruzi* lysate, with Balb/c BMDM showing approximately a 10-fold or 5-fold increase, respectively, and B6 BMDM showing approximately a 3-fold or 1.5-fold increase in *Cyp1a1* induction (**Figure 3A**). Furthermore, the *T. cruzi* lysate acted as a weak agonist of the AhR, inducing *Cyp1a1* expression, and the addition of the AhR-specific antagonist CH223191 inhibited this effect (**Figure 3A**).

In **Figure 2**, we demonstrated higher AhR protein expression in SMC from NI-B6 mice compared to Balb/c mice. However, as shown in **Figure 3**, BMDM from NI-B6 mice exhibited lower *Ahr* mRNA levels than those from Balb/c mice. Interestingly, the stimulation of BMDM from B6 mice with various AhR agonists led to a specific downregulation of *Ahr* mRNA levels, while this effect was not observed in BMDM from Balb/c mice (**Figure 3B**). This response closely mirrored the observed pattern in SMC from infected mice, indicating a similar behavior in both cell types. Furthermore, the Ingenuity Pathway Analysis of RNAseq data consistently demonstrated the down-regulation of genes associated with the AhR pathway in BMDM from B6 mice following *T. cruzi* infection (**Supplementary Figure 2**).

Together, these results suggest that the observed differences in AhR-mediated effects between B6 and Balb/c mice can be attributed not only to variations in AhR expression but also to polymorphisms in the AhR gene and the regulatory mechanisms that control AhR expression.

### Differential AhR agonistic activity in plasma of T. cruzi-infected Balb/c and B6 mice

**Figure 4:**
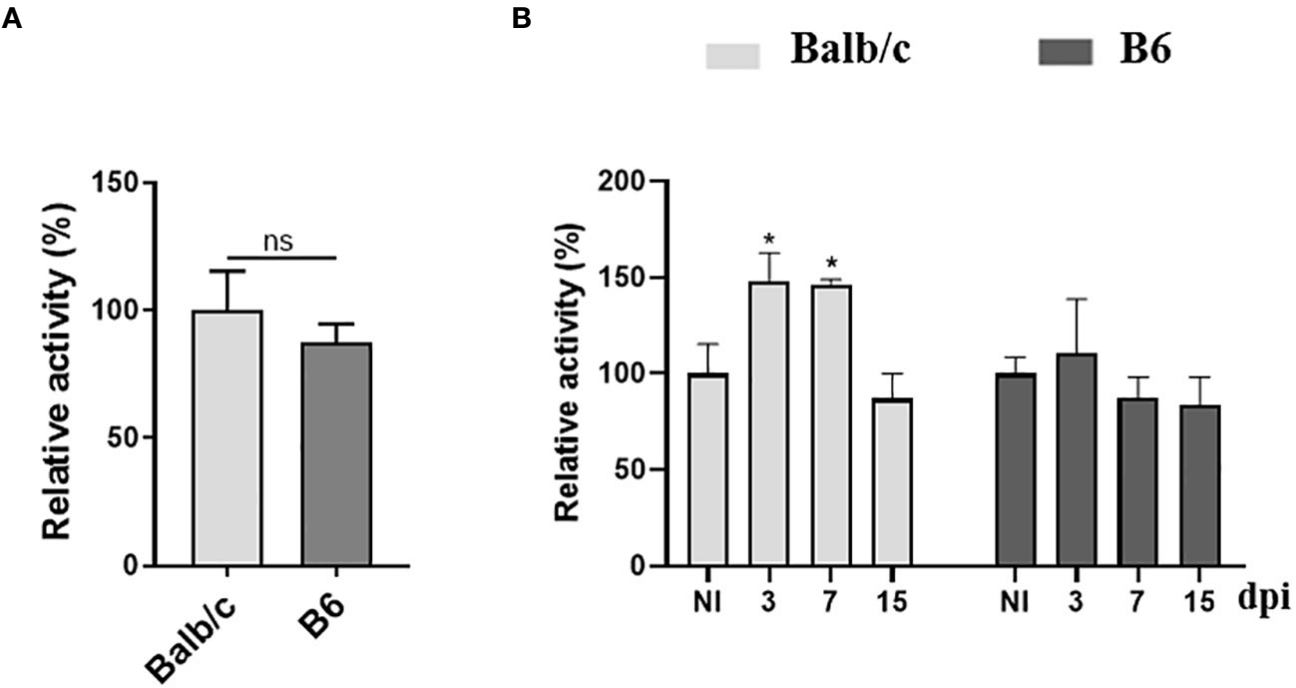
Differential AhR agonistic activity in plasma from T. cruzi-infected Balb/c and B6 mice. The 4T1 murine cell line was transiently transfected with a plasmid containing the DRE sequence from the *Cyp1a1* promoter upstream of a luciferase gene. The cells were then incubated with DMEM containing 10% plasma from non-infected (NI) or T. cruzi-infected Balb/c or B6 mice. **(A)** AhR agonistic activity assessed by luminometry in the plasma samples collected from NI mice. **(B)** AhR agonistic activity assessed by luminometry in the plasma samples collected from infected Balb/c and B6 mice at different times p.i. Relative activity was calculated by dividing firefly luciferase activity (pGud-Luc) by Renilla luciferase activity (pTK-Renilla). Values are presented as means of triplicate measurements. Bars indicate mean ± SD, with n=4 mice per time point. Statistical analysis was performed using Student’s t-test **(A)**, and one-way ANOVA followed by Dunnett’s test **(B)**. The statistical significance was calculated by comparing each time point to the NI of the respective group and is denoted by (*)p<0.05; ns, non-significant **(B)**. The results shown are representative of three independent experiments.

To assess the impact of *T. cruzi* infection on the production of AhR ligands, we measured AhR agonistic activity in the plasma of Balb/c and B6 mice at different time points p.i. using a luciferase expression-based reporter assay as previously described by Rothhammer et al. (45). **Figure 4A** shows that similar levels of AhR agonist activity were detected in the plasma of NI Balb/c and B6 mice. However, interestingly, *T. cruzi* infection led to an increase in the levels of AhR agonist activity in the plasma of Balb/c mice, while no significant increase was observed in those from B6 mice (**Figure 4B**). This early systemic increase in AhR agonist activity in Balb/c mice is consistent with our previous findings demonstrating elevated IDO activity and KYN production in this mouse strain (**Figures 1A, B**).

### Reduced AhR agonistic activity and increased KYN levels in plasma from CCD patients

**Figure 5:**
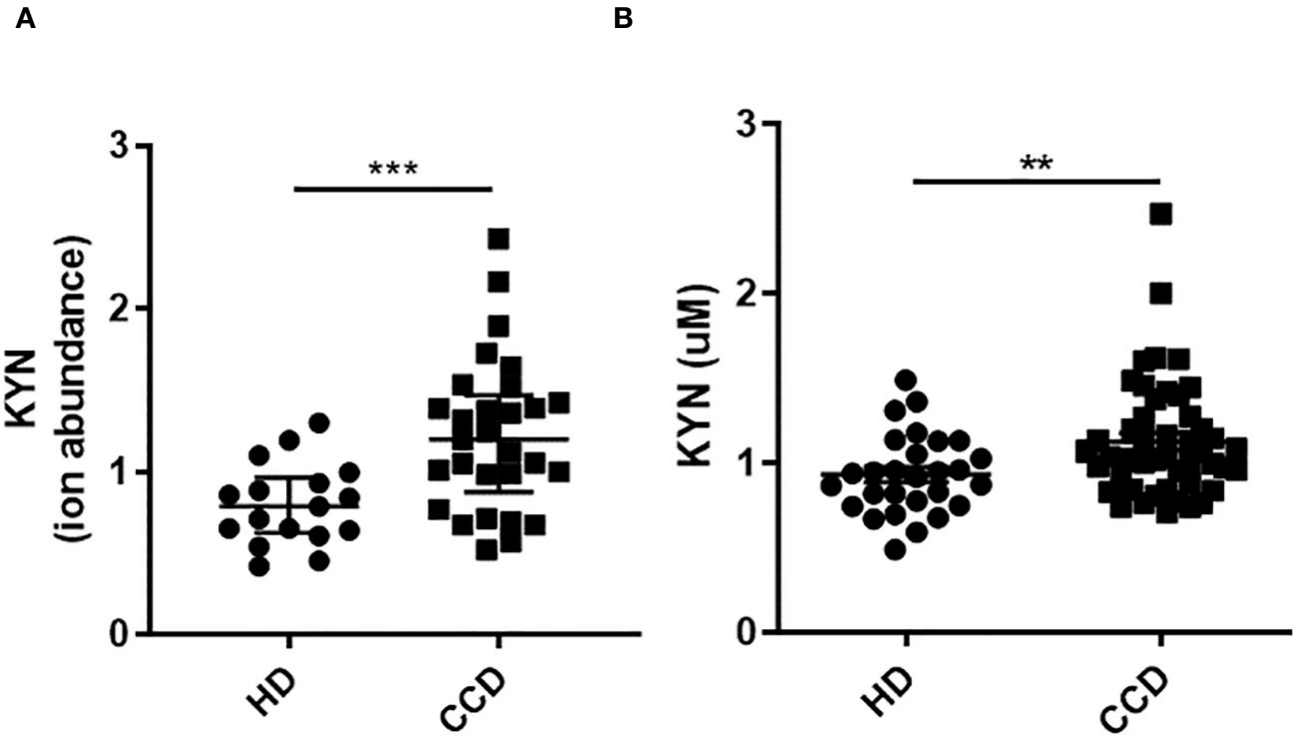
Plasma KYN Levels in CCD Patients and HD. **(A)** Relative Ion Abundance of KYN Determined by LC-MS: The graph illustrates the median values and interquartile range of normalized ion abundance for tryptophan metabolites, highlighting the differences between CCD patients and HD groups. **(B)** KYN Concentration Determined by HPLC: The graph presents the mean ± standard deviation of KYN concentration data. Statistical analysis was performed using the Mann Whitney U-test to determine significant differences. (*)p ≤0.05; (**)p ≤0.01; (***)p ≤0.001; (****)p ≤0.0001.

Investigating the potential up-regulation of IDO activity in Chagas patients following *T. cruzi* infection, we analyzed KYN levels in the plasma of CCD patients using liquid chromatography tandem mass spectrometry (LC-MS) configured on a U-HPLC coupled to an Triple Quadrupole mass spectrometer. These levels were then compared to KYN levels in the plasma of HD. Like the observations in mice, our results showed higher levels of KYN in the plasma of CCD patients compared to the plasma of HD (**Figures 5A, B**).

As mentioned previously, the production of AhR ligands during acute inflammation involves various mechanisms, including the up regulation of IDO enzymatic activity, which generates these anti-inflammatory catabolites. Given the persistent cardiac inflammation observed in Chagas cardiomyopathy (CCC) patients, we hypothesized that it could potentially influence the levels of AhR agonists in the serum. To assess the circulating levels of plasma AhR ligands in patients, we utilized a global AhR-responsive reporter assay. Detailed information regarding the sex and age distribution of patients and controls, as well as the size of the cohort, can be found in **Table 1**.

**Table 1:** Clinical and demographic characteristics of the cohort.

Figure	Cohorte	Females/Male	Age
**1A**	**Control** (17)	**13/4**	**46.52 (39-53)**
	**INF** (29)	**20/9**	**49.48 (41-53)**
**1B**	**Control** (25)	**16/9**	**42.15 (37-50)**
	**IND** (18)	**12/6**	**45.61 (40-53)**
	**CCC** (18)	**12/6**	**46.5 (41-55)**
**1C**	**Control** (17)	**12/5**	**43.37 (41-54)**
	**IND** (15)	**10/5**	**49.61 (40-57)**
	**Mild** (7)	**6/1**	**50.14 (41-55)**
	**Severe** (7)	**5/2**	**48.85 (42-53)**

Each group is denoted by its respective name as displayed in the corresponding figure, with the sample size (n) provided in brackets. Sex: The numbers represent the absolute number of female individuals (biological sex) from left to right, followed by the percentage of females in the group. Age: The average age is indicated, with the 25% and 75% percentiles provided in parentheses. For males, the overall mean age was 46 years, and 41 for females. The prevalent manifestation of Chagas disease was the indeterminate form (53%), followed by the cardiac form (45%). Mild cardiac patients showing any of the following alterations by ECG: incomplete right bundle branch block (47%) or complete (23%), first degree of atrioventricular block (32%) or non-life-threatening arrhythmias (76%). Patients with severe myocarditis, presenting major ECG pathological tracings, that is, complex ventricular arrhythmia (53%) or complete atrioventricular block (68%) and/or congestive heart failure (73%). Additionally, systemic arterial hypertension emerged as a common comorbidity, affecting 53% of the patients.

**Figure 6:**
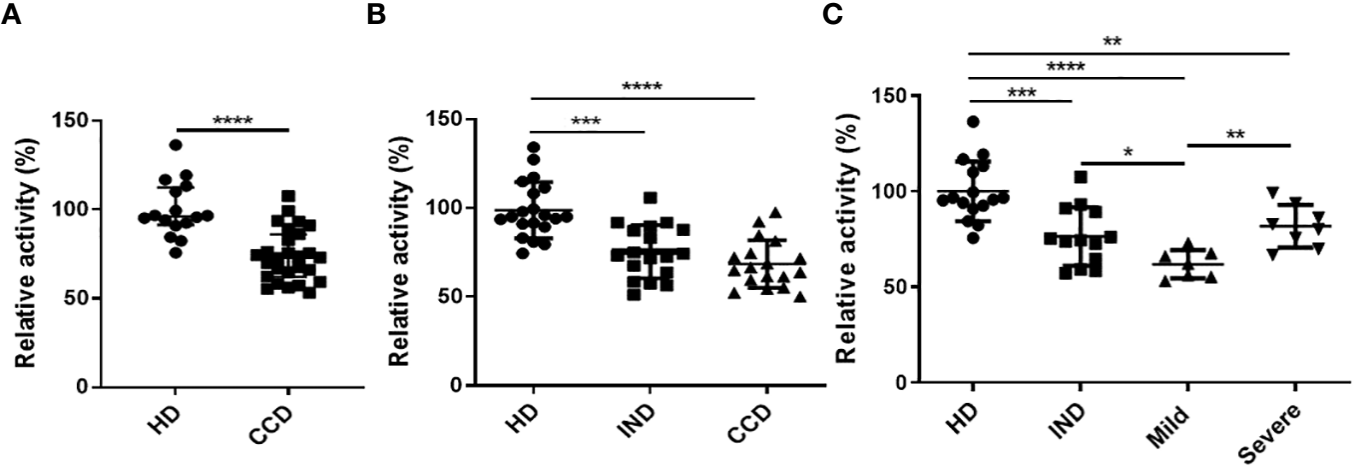
Reduced AhR Agonistic Activity in Plasma from CCD Patients. The HEK293 murine cell line was transiently transfected with a plasmid containing the DRE sequence from the Cyp1a1 promoter placed upstream of a luciferase gene, followed by incubation with DMEM containing 10% plasma from CCD patients or HD. AhR agonistic activity was measured using luminometry. **(A)** Relative AhR agonistic activity in plasma of CCD patients and HD. **(B)** Relative AhR agonistic activity in plasma of HD and CCD patients classified as indeterminate (IND) or presenting chronic Chagas disease cardiomyopathy (CCC). **(C)** Relative AhR agonistic activity in plasma of HD and CCD patients classified as IND and exhibiting mild or severe CCC. P values were calculated using the Mann-Whitney U-test. (*)p ≤0.05; (**)p ≤0.01; (****)p ≤0.0001.

The plasma samples from CCD patients exhibited decreased AhR agonistic activity compared to the HD (**Figure 6A**). To further investigate, we categorized the patients based on the clinical presentation of the disease. The chronic patients were divided into two groups: Indeterminate (IND) and with Chagas disease cardiomyopathy (CCC), as described in the Materials and Methods section. Within the CCC group, patients were further sub-grouped as Severe or Mild based on the severity of their cardiomyopathy.

The levels of AhR agonistic activity in plasma showed no significant differences between the IND and CCC patients (**Figure 6B**). However, a negative trend was observed between the levels of AhR agonistic activity and the presence of cardiomyopathy (**Figure 6B**). These findings, combined with the results obtained from the infected Balb/c and B6 mice (**Figure 4**), suggest a correlation between the level of inflammation during *T. cruzi* infection and reduced AhR agonistic activity.

Interestingly, when analyzing the CCC group based on the presence of Mild or Severe cardiomyopathy, the Severe CCC patients exhibited significantly higher levels of AhR agonistic activity compared to the Mild CCC patients (**Figure 6C**).

### Metabolic profiling in the plasma of CCD patients and HD

It has been reported that in addition to KYN, other Trp catabolites also act as AhR ligands. Therefore, these additional Trp catabolites could potentially contribute to the observed differences in the levels of global AhR agonistic activity between CCD patients and HD.

To investigate whether and how the profiles of Trp catabolites are associated with infection or clinical severity, we conducted a targeted analysis using LC-MS to examine the plasma profiles of Trp metabolites. This analysis was performed on plasma samples obtained from CCD patients (n=30) and age- and sex-matched healthy donors (n=17).

**Figure 7:**
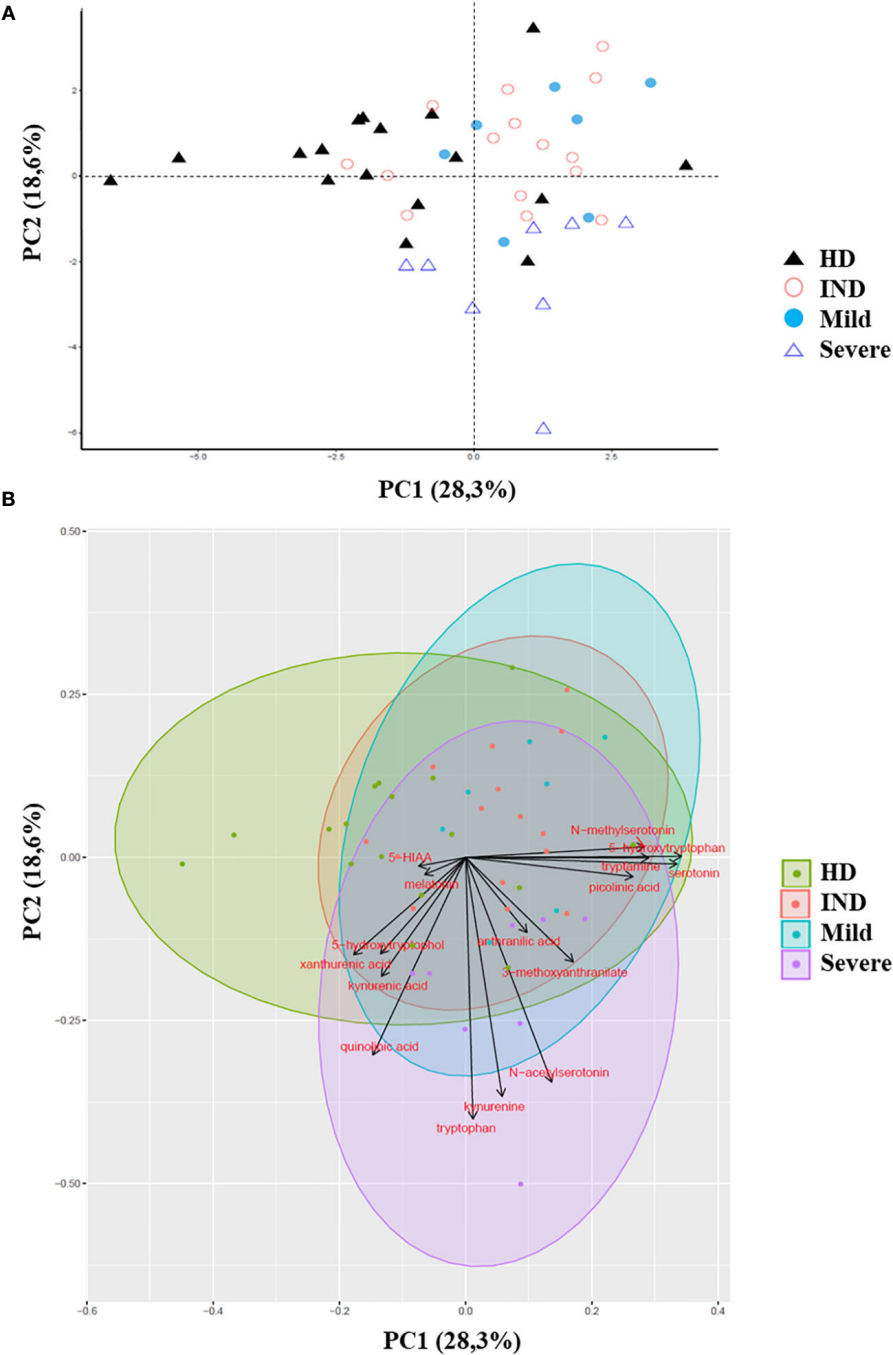
Principal Component Analysis of Trp Metabolites in Plasma of CCD Patients and HD. The principal component analysis (PCA) of Trp metabolites was visualized as follows: **(A)** Score plot graph: The samples are represented by points, with different shapes and colors indicating different groups. **(B)** Biplot graph: The samples are represented by points colored according to their respective groups. The vectors, represented by arrows, demonstrate the direction and magnitude of the contribution of each metabolite to the individual correlations represented by PC1 and PC2.

Remarkably, we observed significant alterations in the plasma levels of multiple Trp metabolites in CCD patients. To assess the overall metabolic differences, we performed a principal component analysis (PCA) on the dataset. The first two principal components (PC1 and PC2) accounted for a cumulative 47% of the total variability observed (**Figure 7**).

The PCA plot revealed distinct clustering patterns between HD and CCD patients. In PC1, HD samples predominantly grouped on the left side of the score plot, while samples from IND, Mild, and Severe groups were primarily located on the right side (**Figure 7A**). Additionally, PC2 reflected the metabolic variation among the CCD patient groups, with samples from Severe patients predominantly found in the lower part of the plot (below 0), while samples from IND and Mild patients were preferentially positioned in the upper part (**Figure 7A**).

To better understand the contribution of specific metabolites to the observed differences between groups, we constructed a biplot graph. In this graph, the length of the vectors represents the extent to which a particular Trp metabolite contributes to the separation between the groups, while the direction and proximity of the vectors to individual samples indicate the presence of the metabolite within each group (**Figure 7B**).

The biplot graph revealed that several Trp metabolites played significant roles in distinguishing between the groups. Based on the vector lengths, the metabolites 5-hydroxytryptophol, xanthurenic acid (XA), kynurenic acid (KYNA), quinolinic acid (QA), Trp, KYN, N-acetylserotonin (NAS), 3-methoxyanthranilate, picolinic acid (PA), serotonin, tryptamine, 5-hydroxytryptophan (5-HTP), and N-methylserotonin were found to contribute substantially to the separation observed between the groups (**Figure 7B**).

**Figure 8:**
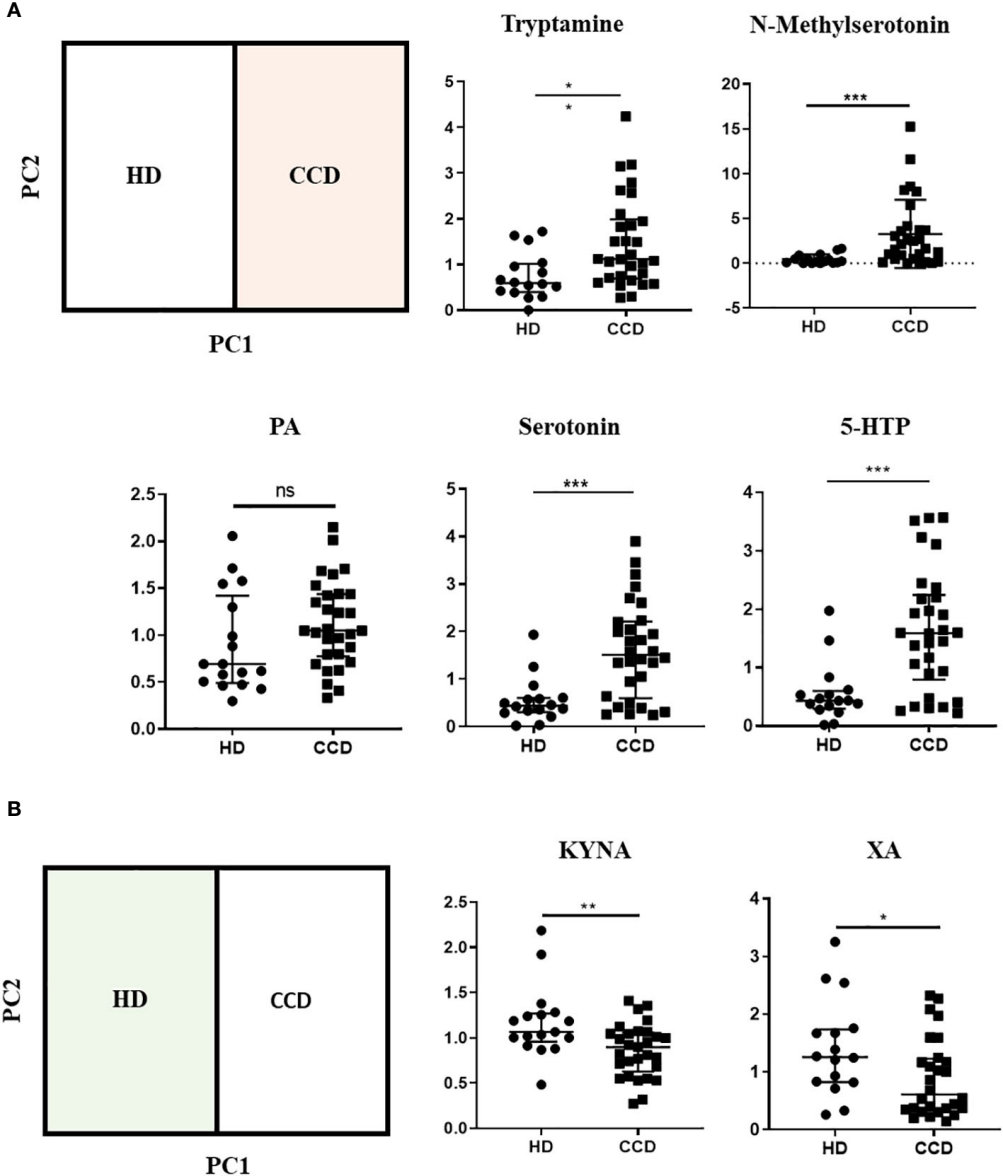
Metabolic Profiles of Trp Metabolites in the Plasma of CCD Patients and HD. **(A)** Relative ion abundance of Tryptamine, N-methylserotonin, PA, Serotonin, and 5-HTP in plasma samples of CCD patients and HD. **(B)** Relative ion abundance of KA and XA in plasma samples of CCD patients and HD. Statistical analysis was performed using the Mann-Whitney U-test to determine statistically significant differences compared to HD plasma samples. (*)p ≤ 0.05; (**)p ≤ 0.01; (***)p ≤ 0.001; ns, non-significant.

To further support our findings, we quantitatively analyzed the normalized ionic abundance of each metabolite. We focused on the main metabolites that contributed to the differences between the samples from HD and CCD patients. We observed that Tryptamine, N-methylserotonin, serotonin, and 5-HTP were significantly increased in the plasma of patients compared to controls (**Figure 8A**). Interestingly, the levels of KYNA and XA, both known AhR agonists, were significantly lower in the plasma of patients compared to HD (**Figure 8B**), which aligns with the reduced AhR agonistic activity detected in the plasma of infected patients (**Figure 6**).

**Figure 9:**
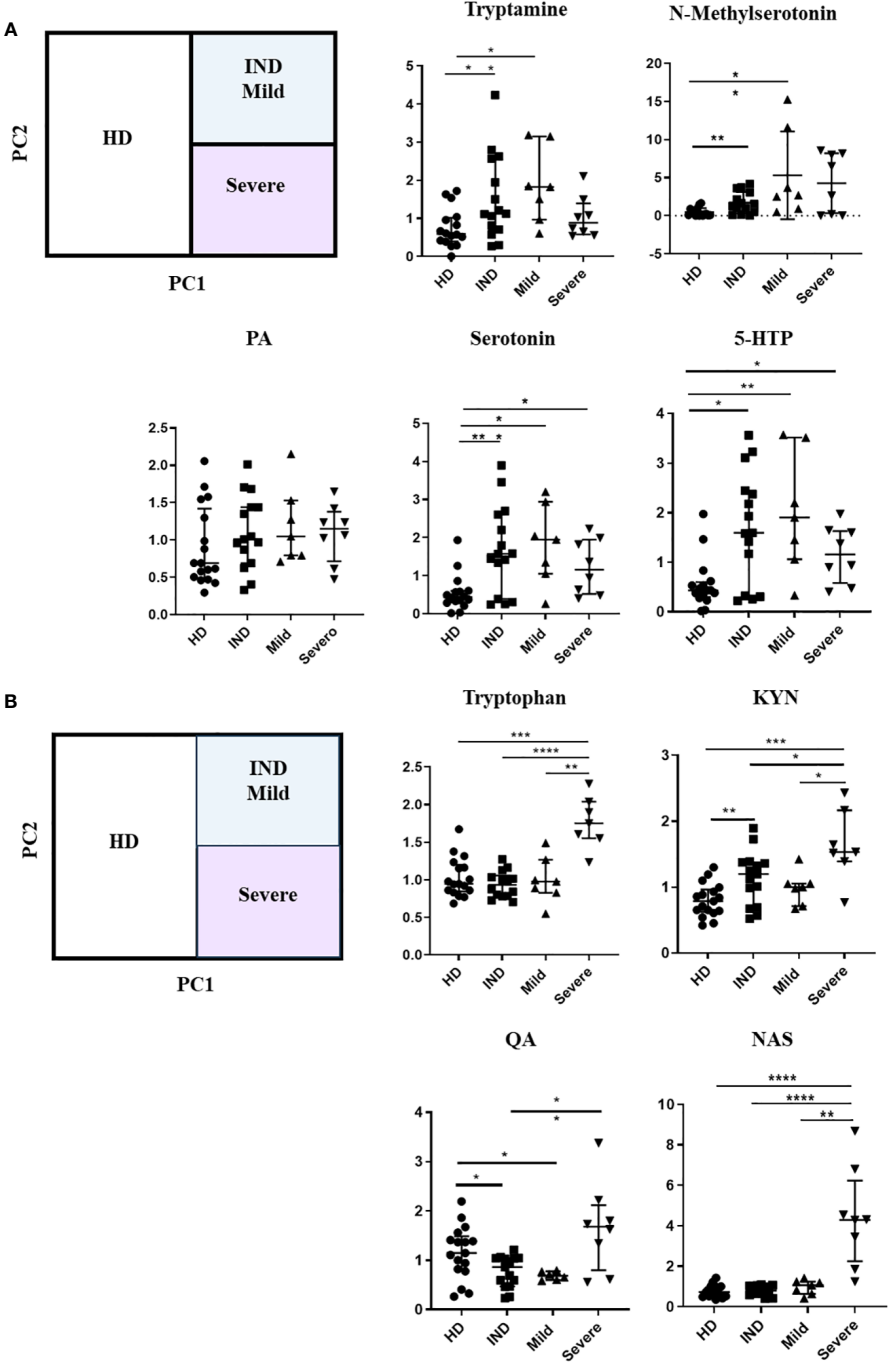
Alterations in Trp Metabolites in Plasma Across Different Stages of Chronic Chagas Disease. **(A)** Relative ion Abundance of Tryptamine, N-methyl serotonin, PA, Serotonin, and 5-HTP, and **(B)** Relative ion abundance of Trp, KYN, QA, and NAS in plasma samples from HD and IND, Mild, and Severe groups of patients. The graphs depict the median values and interquartile range of the normalized ion abundance of Trp metabolites. They highlight the differences in ion abundance between HD vs CCD patients, and Severe vs HD, IND, and Mild groups. Statistical analysis was performed using the Mann-Whitney U-test to determine statistically significant differences compared to HD plasma samples. (*)p ≤ 0.05; (**)p ≤ 0.01; (***)p ≤ 0.001; ns, non-significant.

Subsequently, we investigated the variation in Trp metabolites in plasma across different stages of chronic infection (IND, Mild, and Severe). Our analysis revealed that only serotonin and 5-HTP consistently showed increased levels in all patient groups compared to the HD group (**Figure 9A**). In addition, we did not observe significant differences in the ion abundance of KYNA and XA between the HD and all patient groups (**Supplementary Figure 3**). However, it is important to note that the median values observed of KYNA and XA in the plasma of IND, Mild, and Severe groups were either lower or similar to the values observed in the first quartile (Q1) of the control group. This indicates that, for KYNA and XA, at least 50% of the IND, Mild, and Severe samples exhibited lower ion abundance compared to approximately 75% of the control samples (**Supplementary Figure 3**). The lack of statistical differences may be attributed to data dispersion resulting from the sample size of the Mild and Severe groups. Furthermore, we observed that patients in the Severe group had higher levels of Trp, KYN, QA, and NAS in plasma compared to IND and Mild patients (**Figure 9B**). Specifically, Trp and NAS levels were only increased in the Severe group, with a plasma abundance four times higher than that of the HD, IND, and Mild groups (**Figure 9B**; **Supplementary Figure 4**).

**Figure 10:**
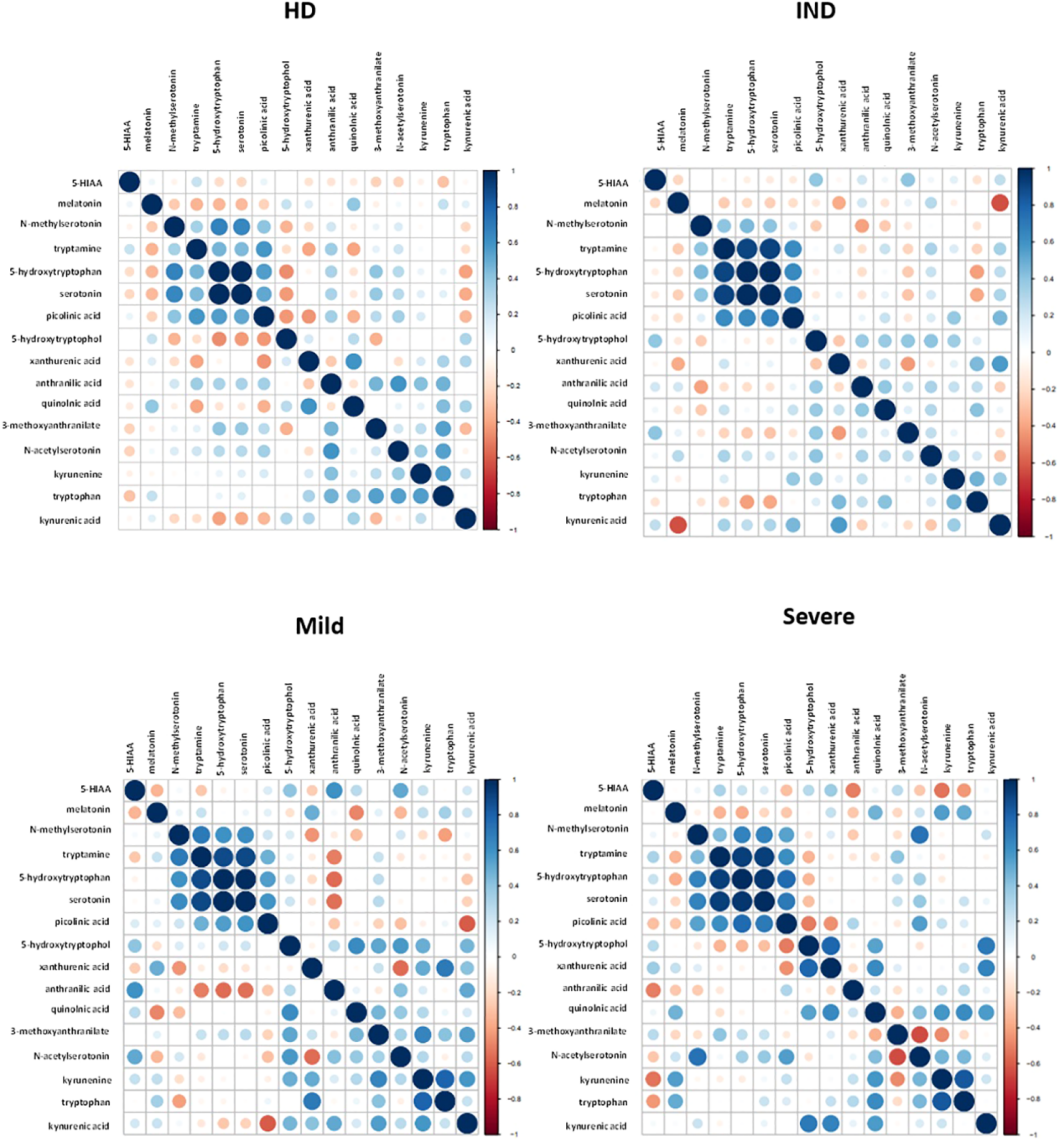
Correlation Plot Between the Relative Ion Abundance of Trp-Related Metabolites in Plasma Samples from HD and CCD Patients. The correlation plot obtained using R displays the relationship between Trp-related metabolites in plasma samples obtained from HD and CCD patients. The CCD patients are further categorized into IND, Mild, and Severe. In the plot, each data point is represented by a circle. The color and size of the circles indicate the strength of the correlation between the metabolites, based on the scale specified in each panel. The color gradient reflects the degree of correlation, with darker shades indicating stronger correlations. Similarly, the size of the circles represents the magnitude of the correlation, with larger circles denoting higher correlation values.

**Figure 11:**
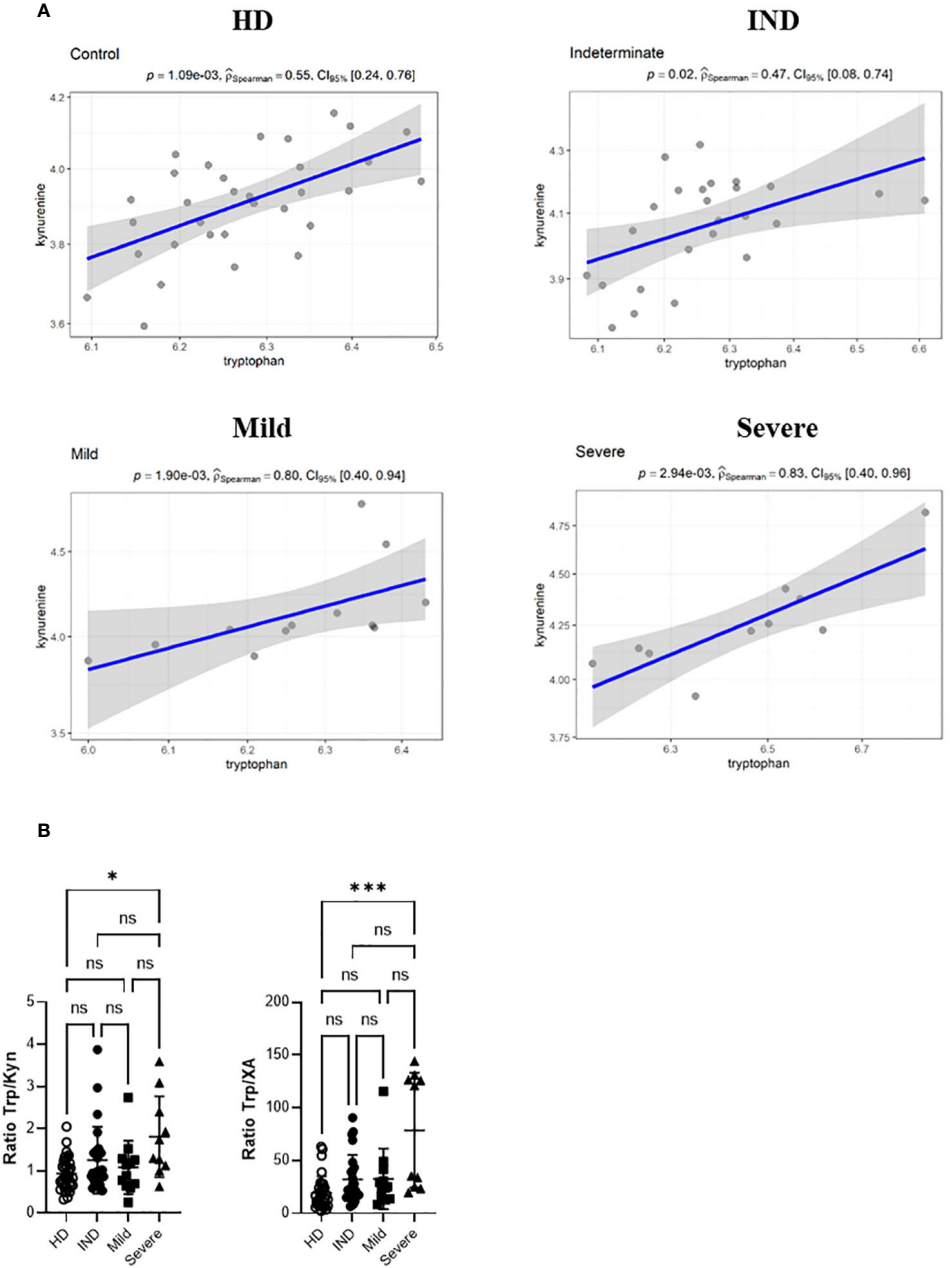
Spearman correlation Between the Relative Ion Abundance of Trp and KYN in Plasma Samples from HD and CCD Patients. **(A)** Spearman correlation scatter plots (linear regression, blue line) with its confidence interval (gray area) for the relative ion abundance of Trp vs KYN in the plasma samples from HD and CCD patients. The CCD patients are further categorized into IND, Mild, and Severe. Spearman correlation coefficient (P_Spearman_) and p associated value is shown. **(B)** Ratio between the ion abundance of Trp and KYN and XA in the plasma samples from HD and CCD patients.

We visualized the correlations between the amounts of metabolites in each group using a correlation plot (**Figure 10**). In the Severe group, Trp levels positively correlated with the three metabolites previously found to be significantly increased in this group: KYN, QA, and NAS (**Figure 10**). **Supplementary Figure 5** displays the correlations. In the Mild group, Trp showed a correlation with KYN, 3-methoxyanthranillate, and XA, while in the IND group, it correlated with KYN (**Figure 10**). Although Trp showed a significant correlation with KYN in all CCD patient groups (**Figure 11A**), in Severe patients the Trp/KYN and Trp/XA ratios were notably different due to significantly higher Trp levels (**Figure 11B**).

These results indicate that *T. cruzi* infection is associated with a distinct profile of Trp metabolites in CCD patients, particularly in those who have developed the severe form of the disease. However, it is not yet clear whether this altered profile is a cause or a consequence of the disease progression. Therefore, we believe that scores of some of these metabolites could serve as suitable biomarkers for disease or clinical severity.

## Discussion

The precise mechanisms involved in the development and progression of cardiac pathology in Chagas Disease have not been fully elucidated. However, there is substantial evidence suggesting that immunoregulatory cytokines play a crucial role in orchestrating the immune response, thereby influencing the development or control of the disease (6–8). During *T. cruzi* infection, B6 mice display an exaggerated Th1 response that is effective in controlling parasite replication. However, similar to CCC patients, B6 mice are unable to regulate the inflammatory response, resulting in severe hepatic damage (15). This is characterized by elevated TNF levels, decreased IL-10 levels, and an inability to expand the Treg population, as observed in comparison to Balb/c mice (15, 16).

We have previously demonstrated the critical role of IDO activity in controlling *T. cruzi* replication, and specifically, the Trp catabolite 3-HK has been shown to inhibit *T. cruzi* replication and induce the generation of Treg Foxp3+ cells when administered *in vivo (* 19*).* Furthermore, the AhR transcription factor, is known to be essential for IDO transcription. It has been reported that various Trp-derived compounds, including certain kynurenines, can act as AhR ligands, leading to AhR activation and the development of a regulatory immune response (22, 34).

In this study, we aimed to investigate the effects of *T. cruzi* infection on AhR expression, as well as AhR-associated features such as IDO activity and Treg cell induction. We also examined the presence of AhR ligands in two mouse strains that exhibit differential susceptibility to developing inflammatory pathology following *T. cruzi* infection. Additionally, as a parallel analysis, we analyzed the expression of AhR ligands and the profiles of Trp-derived metabolites in the plasma of CCD patients without cardiac complications (IND) and those with Mild or Severe CCC, comparing them with HD.

Our findings demonstrate that AhR-dependent features, including IDO activity, KYN levels, and Treg induction, are impaired in *T. cruzi*-infected B6 mice. We observed lower IDO activity in infected B6 mice compared to Balb/c mice, which could be directly attributed to the down-regulation of AhR expression in this particular mouse strain following *T. cruzi* infection.

AhR activity can be regulated by various transcription factors, with one of them being the glucocorticoid receptor (GR) (55, 56). *In vitro* assays conducted on murine hepatoma cell lines have shown that incubation with dexamethasone enhances AhR transcription through a GR-dependent mechanism, leading to an increased response to AhR ligands (56). Moreover, studies have reported that the expression of AhR decreases in the livers of hypophysectomized rats (56, 57). Roggero et al. (58) reported that there is a rapid increase in corticosterone levels during the early stages of *T. cruzi* infection in Balb/c mice. In contrast, B6 mice did not show significant differences in corticosterone levels until three weeks post-infection. Additionally, it has been documented that endogenous glucocorticoids play a crucial role in maintaining a balanced T effector/Treg ratio during *T. cruzi* infection (16). Therefore, the delayed increase in corticosterone levels in the B6 strain of mice could be associated with the lower AhR expression, differential AhR expression kinetics, and the observed imbalance between T effector and Treg cells following *T. cruzi* infection. Furthermore, the expression of TDO, an enzyme involved in the initial step of Trp metabolism in the liver, increases in response to cortisol, potentially leading to lower circulating Trp levels (59). Therefore, not only the differences observed in IDO activity but also a reduction in TDO activity, associated with lower systemic glucocorticoid levels, could explain the decreased concentration of KYN and AhR agonistic activity observed in B6 mice during acute infection.

Furthermore, our observations indicate that the activation of AhR can be influenced by the extent of its expression and by the presence of genetic polymorphisms. We found that ITE and TCDD induce a greater up-regulation of *Cyp1a1* in Balb/c BMDM through the AhR^b2^ allele compared to B6 AhR^b1^. This suggests that the level of AhR expression, which is lower in B6 mice compared to Balb/c mice, and the genetic variations in these strains may impact AhR activation. Recent studies have demonstrated the involvement of AhR mutations and genetic polymorphisms in inflammatory diseases, highlighting their role in modulating AhR activity (60–64). For instance, in mice a single point mutation in AhR (AhRd) reduces its activation in response to TCDD, requiring significantly higher doses to achieve a similar level of activation compared to TCDD high responder strains such as B6 and Balb/c mice (65). Additionally, exposure to diesel exhaust particles, which consist of polycyclic aromatic hydrocarbons and their derivatives, has been shown to result in higher AhR^b2^ (Balb/c) activation compared to AhR^b1^ (B6) (40). It is important to note that AhR polymorphisms not only affect binding affinity but also receptor availability, as structural variations can impact susceptibility to post-translational modifications that regulate AhR concentration within the cell. Changes in the amino acid sequence of AhR, for example, can increase its susceptibility to ubiquitination and subsequent proteasomal degradation (66, 67). This may explain the observed rapid decrease in AhR expression in B6 splenocytes following *T. cruzi* infection.

It has been documented that certain molecules derived from pathogens can function as ligands for AhR (68). In our study, we observed that stimulation of BMDM with *T. cruzi* lysate led to an upregulation of *Cyp1a1* transcription, which was dependent on AhR activation since *Cyp1a1* levels decreased when treated with the AhR antagonist CH223191. However, when we evaluated the AhR agonistic activity of *T. cruzi* lysate using a luciferase reporter assay, we did not observe significant differences compared to the control medium (results not shown). This suggests that detectable AhR ligands were not present in the parasite homogenate, indicating that the upregulation of *Cyp1a1* may have been mediated by the induction of endogenous AhR ligands. It is possible that *T. cruzi* lysate activated IDO activity in BMDM, leading to the generation of AhR ligands or indirect activation of the receptor. Analysis of databases has revealed that the parasite expresses the enzyme kynureninase, which can produce anthranilic acid (AA) or 3-hydroxyanthranilic acid (3-HAA) from KYN and 3-HK, respectively (69). Although the binding of 3-HAA to AhR has not been described, this metabolite can activate AhR through its interaction with NCOA7, a nuclear coactivator expressed in DCs and macrophages, and through the generation of cinnabarinic acid, a metabolite formed from the oxidation of 3-HAA, which has been reported as an AhR ligand (70, 71). Additionally, 3-HAA has been associated with enhancing the immunosuppressive effects of IDO1-expressing DCs and promoting the expansion of Treg cells in an allograft model of small bowel transplantation (72).

In conclusion, our findings demonstrate that *T. cruzi* infection has a differential impact on AhR expression and the generation of AhR ligands, and these differences may be influenced by the host’s genetic component. AhR polymorphisms play a role in determining the magnitude of the ligand response and the susceptibility of AhR to post-translational modifications (66, 67). Given the demonstrated significance of AhR signaling in shaping the regulatory profile of Treg cells (29, 34, 73, 74), the rapid decrease in AhR expression observed in B6-infected mice, coupled with their reduced capacity to generate AhR ligands and activate AhR, may play a role in their impaired ability to induce Treg cells and regulate the heightened inflammatory response observed during *T. cruzi* infection.

Our findings regarding the global agonistic activity of AhR in the plasma of patients with CCC, IND, or HD (**Figure 6**) are consistent with a study by Rothhammer et al. (45), which demonstrated that the overall balance of AhR ligands is lower in the plasma of patients with relapsing-remitting multiple sclerosis (RRMS) compared to that of healthy individuals. Additionally, they found that plasma samples from patients with active RRMS exhibit higher global AhR agonistic activity than those from RRMS-remitting individuals, similar to our observations between the groups with Severe and Mild cardiomyopathy. These results suggest a possible association between the presence of a heightened inflammatory state and lower levels of global AhR agonistic activity. However, the abundance of KYN ions (detected by LC-MS) and the concentration of KYN (measured by HPLC) are higher in the plasma of patients compared to HD, which is consistent with findings from the murine model. Together, these findings indicate that the reduction in global AhR agonistic activity in the plasma of CCD patients may be attributed to a decrease in the production of AhR ligands, different from KYN, during *T. cruzi* infection. In support of the previous conclusion, our findings revealed that the levels of KYNA and XA were lower in CCD patients compared to HD. It is worth noting that these metabolites have the ability to activate human AhR, as demonstrated in a luciferase reporter assay (75). Therefore, the decrease in KYNA and XA levels could potentially contribute to the diminished global agonistic activity detected in the plasma of CCD patients. Furthermore, it is important to note that the ion abundance measurements were obtained through targeted metabolomics, which limited the identification of AhR ligands to a specific group of known metabolites. Additionally, previous studies have shown that intermediate metabolites generated from KYN have the ability to spontaneously activate AhR at picomolar concentrations, whereas activation by KYN itself requires micromolar concentrations. Therefore, the differences in AhR agonistic activity observed between HD and CCD patients may also be influenced by unidentified ligands, metabolites not targeted in this study, and others whose concentrations were below the detection range of the LC-MS method.

A global metabolomic study was conducted on plasma and cardiac tissue samples from Balb/c mice infected with the *T. cruzi* Y strain. The results revealed that approximately one-third of the detected metabolites in the plasma and two-thirds of those in the tissue exhibited differential expression between acutely infected and non-infected animals (76). We further extended this analysis by conducting a unique metabolomic study on the plasma of CCD patients categorized as either IND or with CCC Severe or Mild. Although the majority of Trp undergoes metabolism through the KYN pathway, alterations in metabolites from other Trp pathways were observed in the patients’ plasma (**Supplementary Figure 3**). Notably, among these metabolites, both serotonin and tryptamine were identified as AhR ligands (77, 78).

The results obtained indicate that Trp, KYN, QA, and NAS contribute to the observed differences between the plasma of CCD patients with Severe pathology and the other three groups. Consistent with this, a previous study by Girones et al. (76) reported an increase in KYN levels in both the plasma and cardiac tissue of *T. cruzi*-infected Balb/c mice at day 21 p.i., coinciding with the detection of cardiac pathology in that model. Interestingly, while an increase in Trp levels was observed in cardiac tissue, it was not observed in plasma. Although the higher Trp levels detected in Severe patients could potentially indicate Trp accumulation due to low activity of Trp-degradating enzimes, the increase in Trp-derived metabolites does not support this hypothesis. Trp availability in the plasma is regulated, in part, by the presence of albumin. Inflammatory processes can reduce albumin levels by increasing its fractional catabolic rate, which may lead to increased free-Trp levels in the plasma of Severe patients as a result of albumin reduction caused by higher inflammation levels in this group (79, 80).

In the KYN pathway, modifications in the enzymes involved can disrupt the normal production of metabolites. After Trp degradation mediated by IDO or TDO, which generates KYN, there are two main catabolic branches. One branch is mediated by kynurenine 3-monooxygenase (KMO) and kynureninase (KYNU), leading to the formation of 3-HK, 3-HAA, and QA. The other branch is controlled by kynurenine aminotransferase (KAT), which produces KYNA and XA (see **Supplementary Figure 3**) (75, 81). KMO and KYNU are more susceptible to regulation by inflammatory stimuli, with their expression increasing in the presence of lipopolysaccharide (LPS), interferon-gamma (IFN-γ), and interleukin-1 beta (IL-1β), which could explain the observed increase in QA levels in the Severe group (82). On the other hand, KAT expression is decreased or unaffected in inflammatory contexts (82), which is consistent with the lower levels of KYNA and XA detected in CCD samples.

NAS, synthesized from serotonin, is primarily known for its antidepressant and neuroprotective effects and plays a role in melatonin generation (83). Although there are no specific reports associating NAS with cardiac functionality, serotonin and melatonin have been linked to cardiac function. For instance, increased serotonin levels have been associated with hypertension, while melatonin provides resistance against ischemic reperfusion damage and protects against myocardial inflammation induced by intermittent chronic hypoxia and fibrosis, among other effects (84, 85). Interestingly, we observed a tendency of melatonin reduction as cardiac pathology worsens, and this decrease may be related to lower melatonin generation from NAS, considering the higher levels of NAS detected in the Severe group.

To the best of our knowledge, there are no other studies reporting Trp metabolite levels in patients with CCD disease using LC-MS. This study demonstrates that during chronic *T. cruzi* infection, there is dysregulation of Trp metabolic pathways, specifically serotonin, tryptamine, and KYN, which is reflected in the plasma composition. Additionally, we have identified NAS as a potential biomarker of severity; however, further investigation is required to determine whether NAS elevation is exclusive to Chagas cardiomyopathy or if it is also increased in other cardiopathies of different etiology. Changes in Trp metabolite expression have been associated with various conditions, including inflammatory bowel disease, CNS-related pathologies such as Huntington’s, Parkinson’s, and Alzheimer’s diseases, as well as cardiovascular disease and its mortality (81, 86, 87). Therefore, understanding the mechanisms responsible for the imbalance in Trp metabolites is crucial for the development of new therapeutic strategies, such as designing specific enzyme inhibitors and identifying disease biomarkers (88).

## Data availability statement

The data presented in the study are deposited in the Gene Expression Omnibus (GEO) and MetaboLights repositories, accession number Code GSE244478 and MTBLS8390, respectively.

## Ethics statement

The studies involving humans were approved by Ethics Committee of the Facultad de Medicina de la Universidad Nacional de Rosario, Santa Fe, Argentina (RES N°: 666/2015). The studies were conducted in accordance with the local legislation and institutional requirements. The participants provided their written informed consent to participate in this study. The animal study was approved by Animal Care and Use Committee of the Facultad de Ciencias Químicas, Universidad Nacional de Córdoba (Approval Number HCD 743/18). The study was conducted in accordance with the local legislation and institutional requirements.

## Author contributions

LA: Data curation, Formal Analysis, Investigation, Methodology, Validation, Visualization, Conceptualization, Writing – original draft, Writing – review & editing. XV: Visualization, Conceptualization, Data curation, Formal Analysis, Investigation, Methodology, Writing – original draft, Writing – review & editing. JQ: Conceptualization, Data curation, Formal Analysis, Investigation, Methodology, Writing – original draft. MB: Conceptualization, Data curation, Formal Analysis, Methodology, Writing – original draft. CK: Investigation, Data curation, Formal Analysis, Methodology, Visualization, Writing – original draft. MH: Data curation, Formal Analysis, Methodology, Visualization, Writing – original draft. LF: Data curation, Writing – review & editing, Formal Analysis, Methodology, Resources, Writing – original draft. JP: Supervision, Writing – original draft, Formal Analysis, Methodology, Resources. CC: Data curation, Methodology, Supervision, Writing – original draft, Resources, Software, Visualization. MT: Data curation, Formal Analysis, Methodology, Supervision, Validation, Writing – original draft. JB: Data curation, Formal Analysis, Methodology, Supervision, Writing – original draft, Resources, Validation. MT: Visualization, Writing – original draft, Writing – review & editing, Data curation, Formal Analysis, Methodology, Supervision. FQ: Resources, Visualization, Writing – original draft, Writing – review & editing, Funding acquisition, Project administration, Validation. AP: Conceptualization, Data curation, Methodology, Resources, Supervision, Visualization, Writing – original draft, Writing – review & editing. CM: Conceptualization, Funding acquisition, Project administration, Supervision, Writing – original draft, Writing – review & editing.

## References

[1] Salvatella R Irabedra P Sánchez D Castellanos LG Espinal M. South-south cooperation for Chagas disease. Lancet (2013) 382(9890):395–6. doi: 10.1016/S0140-6736(13)61671-2 23911378

[2] Schofield CJ Jannin J Salvatella R. The future of Chagas disease control. Trends parasitol (2006) 22(12):583–8. doi: 10.1016/j.pt.2006.09.011 17049308

[3] Moretti E Basso B Cervetta L Brigada A Barbieri G. Patterns of cytokines and soluble cellular receptors in the sera of children with acute Chagas' disease. Clin Diagn Lab Immunol (2002) 9(6):1324–7. doi: 10.1128/CDLI.9.6.1324-1327.2002 PMC130093 12414768

[4] Abel LC Rizzo LV Ianni B Albuquerque F Bacal F Carrara D. Chronic Chagas' disease cardiomyopathy patients display an increased IFN-γ response to Trypanosoma cruzi infection. J autoimmunity (2001) 17(1):99–107. doi: 10.1006/jaut.2001.0523 11488642

[5] Gomes JAS Bahia-Oliveira LMG Rocha MOC Martins-Filho OA Gazzinelli G Correa-Oliveira R. Evidence that development of severe cardiomyopathy in human chagas' Disease is due to a th1-specific immune response. Infection Immunity (2003) 71(3):1185–93. doi: 10.1128/IAI.71.3.1185-1193.2003 PMC148818 12595431

[6] Araujo FF Gomes J Rocha M Williams-Blangero S Pinheiro VM Morato M. Potential role of CD4+ CD25HIGH regulatory T cells in morbidity in Chagas disease. Front biosci: J virtual library (2006) 12:2797–806. doi: 10.2741/2273 17485260

[7] da Silveira AB de AraÛjo FF Freitas MAR Gomes JAS Chaves AT de Oliveira EC. Characterization of the presence and distribution of Foxp3+ cells in chagasic patients with and without megacolon. Hum Immunol (2009) 70(1):65–7. doi: 10.1016/j.humimm.2008.10.015 19022313

[8] de Araujo FF Vitelli-Avelar DM Teixeira-Carvalho A Antas PR Assis Silva Gomes J Sathler-Avelar R. Regulatory T cells phenotype in different clinical forms of Chagas' disease. PloS Negl Trop Dis (2011) 5(5):e992. doi: 10.1371/journal.pntd.0000992 21655351 PMC3104959

[9] Pérez AR Silva-Barbosa SD Berbert LR Revelli S Beloscar J Savino W. Immunoneuroendocrine alterations in patients with progressive forms of chronic Chagas disease. J Neuroimmunol (2011) 235(1):84–90. doi: 10.1016/j.jneuroim.2011.03.010 21496931

[10] de Lourdes Higuchi M Gutierrez PS Aiello VD Palomino S Bocchi E Kalil J. Immunohistochemical characterization of infiltrating cells in human chronic chagasic myocarditis: comparison with myocardial rejection process. Virchows Archiv A. (1993) 423(3):157–60. doi: 10.1007/BF01614765 7901937

[11] Rocha Rodrigues DB dos Reis MA Romano A Pereira SA Teixeira Vde P Tostes S Jr.. *In situ* expression of regulatory cytokines by heart inflammatory cells in Chagas' disease patients with heart failure. Clin Dev Immunol (2012) 2012:361730. doi: 10.1155/2012/361730 22811738 PMC3397162

[12] Cunha-Neto E Dzau VJ Allen PD Stamatiou D Benvenutti L Higuchi ML. Cardiac gene expression profiling provides evidence for cytokinopathy as a molecular mechanism in Chagas' disease cardiomyopathy. Am J pathol (2005) 167(2):305–13. doi: 10.1016/S0002-9440(10)62976-8 PMC1603558 16049318

[13] Reis MM HIGUCHI M Benvenuti L Aiello Vd Sampaio Gutierrez P Bellotti G. An in *situ* quantitative immunohistochemical study of cytokines and IL-2R+ in chronic human chagasic myocarditis: Correlation with the presence of myocardial Trypanosoma cruzi antigens. Clin Immunol immunopathol (1997) 83(2):165–72. doi: 10.1006/clin.1997.4335 9143377

[14] Argüello RJ Vigliano C Cabeza-Meckert P Viotti R Garelli F Favaloro LE. Presence of antigen-experienced T cells with low grade of differentiation and proliferative potential in chronic Chagas disease myocarditis. PloS Negl Trop Dis (2014) 8(8):e2989. doi: 10.1371/journal.pntd.0002989 25144227 PMC4140664

[15] Roggero E Perez A Tamae-Kakazu M Piazzon I Nepomnaschy I Wietzerbin J. Differential susceptibility to acute Trypanosoma cruzi infection in BALB/c and C57BL/6 mice is not associated with a distinct parasite load but cytokine abnormalities. Clin Exp Immunol (2002) 128(3):421–8. doi: 10.1046/j.1365-2249.2002.01874.x PMC1906265 12067296

[16] González FB Villar SR Fernández Bussy R Martin GH Pérol L Manarin R. Immunoendocrine dysbalance during uncontrolled T. cruzi infection is associated with the acquisition of a Th-1-like phenotype by Foxp3(+) T cells. Brain behavior immunity (2015) 45:219–32. doi: 10.1016/j.bbi.2014.11.016 PMC7126853 25483139

[17] Araujo Furlan CL Tosello Boari J Rodriguez C Canale FP Fiocca Vernengo F Boccardo S. Limited foxp3(+) regulatory T cells response during acute trypanosoma cruzi infection is required to allow the emergence of robust parasite-specific CD8(+) T cell immunity. Front Immunol (2018) 9:2555. doi: 10.3389/fimmu.2018.02555 30455700 PMC6230662

[18] Mellor A. Indoleamine 2,3 dioxygenase and regulation of T cell immunity. Biochem Biophys Res Commun (2005) 338(1):20–4. doi: 10.1016/j.bbrc.2005.08.232 16157293

[19] Knubel CP Martinez FF Fretes RE Lujan CD Theumer MG Cervi L. Indoleamine 2,3-dioxigenase (IDO) is critical for host resistance against Trypanosoma cruzi. FASEB J (2010) 24(8):2689–701. doi: 10.1096/fj.09-150920 20233946

[20] Knubel CP Martinez FF Acosta Rodriguez EV Altamirano A Rivarola HW Diaz Luja¡n C. 3-hydroxy kynurenine treatment controls T. cruzi replication and the inflammatory pathology preventing the clinical symptoms of chronic chagas disease. PloS One (2011) 6(10):e26550. doi: 10.1371/journal.pone.0026550 22028903 PMC3197528

[21] Knubel CP Insfran C Martinez FF Diaz Lujan C Fretes RE Theumer MG. 3-Hydroxykynurenine, a Tryptophan Metabolite Generated during the Infection, Is Active Against Trypanosoma cruzi. ACS Medicinal Chem Letters (2017) 8(7):757–61. doi: 10.1021/acsmedchemlett.7b00169 PMC5512135 28740612

[22] Nguyen NT Kimura A Nakahama T Chinen I Masuda K Nohara K. Aryl hydrocarbon receptor negatively regulates dendritic cell immunogenicity *via* a kynurenine-dependent mechanism. PNAS (2010) 107(46):19961–6. doi: 10.1073/pnas.1014465107 PMC2993339 21041655

[23] Kaiser H Parker E Hamrick MW. Kynurenine signaling through the aryl hydrocarbon receptor: Implications for aging and healthspan. (1873-6815 (Electronic)). Exp Gerontol (2020) 130:110797. doi: 10.1016/j.exger.2019.110797 31786316 PMC7899131

[24] Quintana FJ Sherr DH. Aryl hydrocarbon receptor control of adaptive immunity. Pharmacol Rev (2013) 65(4):1148–61. doi: 10.1124/pr.113.007823 PMC3799235 23908379

[25] Kerkvliet NI. AHR-mediated immunomodulation: The role of altered gene transcription. Biochem Pharmacol (2009) 77(4):746–60. doi: 10.1016/j.bcp.2008.11.021 PMC2662368 19100241

[26] Ishihara YA-O Kado SY Hoeper C Harel S Vogel CFA. Role of NF-kB relB in aryl hydrocarbon receptor-mediated ligand specific effects. Int J Mol Sci (2019) 20(11):2652. doi: 10.3390/ijms20112652 31151139 PMC6600526

[27] Kimura A Naka T Nohara K Fujii-Kuriyama Y Kishimoto T. Aryl hydrocarbon receptor regulates Stat1 activation and participates in the development of Th17 cells. PNAS (2008) 105(28):9721–6. doi: 10.1073/pnas.0804231105 PMC2474493 18607004

[28] Vogel CFA Sciullo E Li W Wong P Lazennec G Matsumura F. RelB, a new partner of aryl hydrocarbon receptor-mediated transcription. Mol Endocrinol (2007) 21(12):2941–55. doi: 10.1210/me.2007-0211 PMC2346533 17823304

[29] Quintana F Basso A Iglesias A Korn T Farez M Bettelli E. Control of T(reg) and T(H)17 cell differentiation by the aryl hydrocarbon receptor. Nature (2008) 453(7191):65–71. doi: 10.1038/nature06880 18362915

[30] Veldhoen M Hirota K Westendorf A Buer J Dumoutier L Renauld J. The aryl hydrocarbon receptor links TH17-cell-mediated autoimmunity to environmental toxins. Nature (2008) 453(7191):106–9. doi: 10.1038/nature06881 18362914

[31] Nguyen L Bradfield C. The search for endogenous activators of the aryl hydrocarbon receptor. Chem Res toxicol (2008) 21(1):102–16. doi: 10.1021/tx7001965 PMC2572005 18076143

[32] Opitz C Litzenburger U Sahm F Ott M Tritschler I Trump S. An endogenous tumour-promoting ligand of the human aryl hydrocarbon receptor. Nature (2011) 478(7368):197–203. doi: 10.1038/nature10491 21976023

[33] Fallarino F Grohmann U You S McGrath BC Cavener DR Vacca C. Tryptophan catabolism generates autoimmune-preventive regulatory T cells. Transpl Immunol (2006) 17(1):58–60. doi: 10.1016/j.trim.2006.09.017 17157218

[34] Mezrich JD Fechner JH Zhang X Johnson BP Burlingham WJ Bradfield CA. An interaction between kynurenine and the aryl hydrocarbon receptor can generate regulatory T cells. J Immunol (2010) 185(6):3190–8. doi: 10.4049/jimmunol.0903670 PMC2952546 20720200

[35] Ambrosio LF Insfran C Volpini X Acosta Rodriguez E Serra HM Quintana FJ. Role of aryl hydrocarbon receptor (AhR) in the regulation of immunity and immunopathology during trypanosoma cruzi infection. Front Immunol (2019) 10:631. doi: 10.3389/fimmu.2019.00631 30984194 PMC6450169

[36] Julliard W Fechner JH Mezrich JD. The aryl hydrocarbon receptor meets immunology: friend or foe? A little of both. Front Immunol (2014) 5:458. doi: 10.3389/fimmu.2014.00458 25324842 PMC4183121

[37] Gargaro M Pirro M Romani R Zelante T Fallarino F. Aryl hydrocarbon receptor–dependent pathways in immune regulation. Am J Transplantation (2016) 16(8):2270–6. doi: 10.1111/ajt.13716 26751261

[38] Chitrala KN Yang X Nagarkatti P Nagarkatti M. Comparative analysis of interactions between aryl hydrocarbon receptor ligand binding domain with its ligands: a computational study. BMC Struct Biol (2018) 18(1):15. doi: 10.1186/s12900-018-0095-2 30522477 PMC6282305

[39] Poland A Glover E. Characterization and strain distribution pattern of the murine Ah receptor specified by the Ahd and Ahb-3 alleles. Mol Pharmacol (1990) 38(3):306–12. 2169579

[40] Izawa H Kohara M Watanabe G Taya K Sagai M. Effects of diesel exhaust particles on the male reproductive system in strains of mice with different aryl hydrocarbon receptor responsiveness. J Reprod Dev (2007) 53(6):1191–7. doi: 10.1262/jrd.19114 17827877

[41] Zuniga E Motran C Montes CL Diaz FL Bocco JL Gruppi A. Trypanosoma cruzi-induced immunosuppression: B cells undergo spontaneous apoptosis and lipopolysaccharide (LPS) arrests their proliferation during acute infection. Clin Exp Immunol (2000) 119(3):507–15. doi: 10.1046/j.1365-2249.2000.01150.x PMC1905583 10691924

[42] Hsu YM Zhang Y You Y Wang D Li H Duramad O. The adaptor protein CARD9 is required for innate immune responses to intracellular pathogens. Nat Immunol (2007) 8(2):198–205. doi: 10.1038/ni1426 17187069

[43] Motran CC Diaz FL Montes CL Bocco JL Gruppi A. *In vivo* expression of recombinant pregnancy-specific glycoprotein 1a induces alternative activation of monocytes and enhances Th2-type immune response. Eur J Immunol (2003) 33(11):3007–16. doi: 10.1002/eji.200323993 14579269

[44] Volpini X Ambrosio LF Fozzatti L Insfran C Stempin CC Cervi L. Trypanosoma cruzi exploits wnt signaling pathway to promote their intracellular replication in macrophages. Front Immunol (2018) 9(859). doi: 10.3389/fimmu.2018.00859 PMC5930390 29743880

[45] Rothhammer V Borucki DM Sanchez MIG Mazzola MA Hemond CC Regev K. Dynamic regulation of serum aryl hydrocarbon receptor agonists in MS. Neurology-Neuroimmunol Neuroinflamm (2017) 4(4):e359. doi: 10.1212/NXI.0000000000000359 PMC5473958 28642887

[46] Kudo Y Boyd CA. Human placental indoleamine 2,3-dioxygenase: cellular localization and characterization of an enzyme preventing fetal rejection. Biochim Biophys Acta (2000) 1500(1):119–24. doi: 10.1016/S0925-4439(99)00096-4 10564724

[47] Wikoff WR Anfora AT Liu J Schultz PG Lesley SA Peters EC. Metabolomics analysis reveals large effects of gut microflora on mammalian blood metabolites. Proc Natl Acad Sci (2009) 106(10):3698–703. doi: 10.1073/pnas.0812874106 PMC2656143 19234110

[48] Zelante T Iannitti RG Cunha C De Luca A Giovannini G Pieraccini G. Tryptophan catabolites from microbiota engage aryl hydrocarbon receptor and balance mucosal reactivity *via* interleukin-22. Immunity (2013) 39(2):372–85. doi: 10.1016/j.immuni.2013.08.003 23973224

[49] Vogel CF Goth SR Dong B Pessah IN Matsumura F. Aryl hydrocarbon receptor signaling mediates expression of indoleamine 2,3-dioxygenase. Biochem Biophys Res Commun (2008) 375(3):331–5. doi: 10.1016/j.bbrc.2008.07.156 PMC2583959 18694728

[50] Munn DH Sharma MD Baban B Harding HP Zhang Y Ron D. GCN2 kinase in T cells mediates proliferative arrest and anergy induction in response to indoleamine 2,3-dioxygenase. Immunity (2005) 22(5):633–42. doi: 10.1016/j.immuni.2005.03.013 15894280

[51] Terness P Bauer TM Rose L Dufter C Watzlik A Simon H. Inhibition of allogeneic T cell proliferation by indoleamine 2,3-dioxygenase-expressing dendritic cells: mediation of suppression by tryptophan metabolites. J Exp Med (2002) 196(4):447–57. doi: 10.1084/jem.20020052 PMC2196057 12186837

[52] Fallarino F Grohmann U Vacca C Bianchi R Orabona C Spreca A. T cell apoptosis by tryptophan catabolism. Cell Death Differ (2002) 9(10):1069–77. doi: 10.1038/sj.cdd.4401073 12232795

[53] Mellor A Munn D. IDO expression by dendritic cells: tolerance and tryptophan catabolism. Nat Rev Immunol (2004) 4(10):762–74. doi: 10.1038/nri1457 15459668

[54] Hassanain HH Chon SY Gupta SL. Differential regulation of human indoleamine 2,3-dioxygenase gene expression by interferons-gamma and -alpha. Analysis of the regulatory region of the gene and identification of an interferon-gamma-inducible DNA-binding factor. J Biol Chem (1993) 268(7):5077–84. doi: 10.1093/infdis/jis280 8444884

[55] Gutiérrez-Vázquez C Quintana FJ. Regulation of the immune response by the aryl hydrocarbon receptor. Immunity (2018) 48(1):19–33. doi: 10.1016/j.immuni.2017.12.012 29343438 PMC5777317

[56] Bielefeld KA Lee C Riddick DS. Regulation of aryl hydrocarbon receptor expression and function by glucocorticoids in mouse hepatoma cells. Drug Metab Dispos (2008) 36(3):543–51. doi: 10.1124/dmd.107.019703 18086832

[57] Timsit YE Chia FS-C Bhathena A Riddick DS. Aromatic hydrocarbon receptor expression and function in liver of hypophysectomized male rats. Toxicol Appl Pharmacol (2002) 185(2):136–45. doi: 10.1006/taap.2002.9526 12490138

[58] Roggero E Perez AR Tamae-Kakazu M Piazzon I Nepomnaschy I Besedovsky HO. Endogenous glucocorticoids cause thymus atrophy but are protective during acute Trypanosoma cruzi infection. J Endocrinol (2006) 190(2):495–503. doi: 10.1677/joe.1.06642 16899582

[59] Oxenkrug GF. Tryptophan–kynurenine metabolism as a common mediator of genetic and environmental impacts in major depressive disorder: the serotonin hypothesis revisited 40 years later. Israel J Psychiatry related Sci (2010) 47(1):56. PMC3021918 20686200

[60] Lamas B Richard ML Leducq V Pham HA-O Michel ML Da Costa G. CARD9 impacts colitis by altering gut microbiota metabolism of tryptophan into aryl hydrocarbon receptor ligands. Nat Med (2016) 22(6):598–605. doi: 10.1038/nm.4102 27158904 PMC5087285

[61] Gunton JE Kulkarni RN Yim S Okada T Hawthorne WJ Tseng Y-H. Loss of ARNT/HIF1β Mediates altered gene expression and pancreatic-islet dysfunction in human type 2 diabetes. Cell (2005) 122(3):337–49. doi: 10.1016/j.cell.2005.05.027 16096055

[62] Yamamoto Y Kiyohara C Ogata-Suetsugu S Hamada N Nakanishi Y. Association between genetic polymorphisms involved in the hypoxia-inducible factor pathway and lung cancer risk: a case–control study in Japan. Asia-Pacific J Clin Oncol (2017) 13(3):234–42. doi: 10.1111/ajco.12640 27981753

[63] Huang S Shui X He Y Xue Y Li J Li G. AhR expression and polymorphisms are associated with risk of coronary arterial disease in Chinese population. Sci Rep (2015) 5:8022. doi: 10.1038/srep08022 25620626 PMC4306136

[64] Wang XW Li K Guo S Qiang HN Liu L Song P. The association of functional polymorphisms in the aryl hydrocarbon receptor (AHR) gene with the risk of vitiligo in Han Chinese populations. Br J Dermatol (2012) 166(5):1081–7. doi: 10.1111/j.1365-2133.2011.10798.x 22211302

[65] Poland A Palen D Glover E. Analysis of the four alleles of the murine aryl hydrocarbon receptor. Mol Pharmacol (1994) 46(5):915–21. 7969080

[66] Pollenz RS. The mechanism of AH receptor protein down-regulation (degradation) and its impact on AH receptor-mediated gene regulation. Chemico-biological interactions (2002) 141(1-2):41–61. doi: 10.1016/S0009-2797(02)00065-0 12213384

[67] Aftabi Y Colagar AH Mehrnejad F. An in silico approach to investigate the source of the controversial interpretations about the phenotypic results of the human AhR-gene G1661A polymorphism. J Theor Biol (2016) 393:1–15. doi: 10.1016/j.jtbi.2016.01.001 26776670

[68] Moura-Alves P Faé K Houthuys E Dorhoi A Kreuchwig A Furkert J. AhR sensing of bacterial pigments regulates antibacterial defence. Nature (2014) 512(7515):387–92. doi: 10.1038/nature13684 25119038

[69] Ecco G Vernal J Razzera G Tavares C Serpa VI Arias S. Initial characterization of a recombinant kynureninase from Trypanosoma cruzi identified from an EST database. Gene (2009) 448(1):1–6. doi: 10.1016/j.gene.2009.08.007 19699281

[70] Lowe MM Mold JE Kanwar B Huang Y Louie A Pollastri MP. Identification of cinnabarinic acid as a novel endogenous aryl hydrocarbon receptor ligand that drives IL-22 production. PloS One (2014) 9(2):e87877. doi: 10.1371/journal.pone.0087877 24498387 PMC3912126

[71] Gargaro M Vacca C Massari S Scalisi G Manni G Mondanelli G. Engagement of nuclear coactivator 7 by 3-hydroxyanthranilic acid enhances activation of aryl hydrocarbon receptor in immunoregulatory dendritic cells. Front Immunol (2019) 10:1973. doi: 10.3389/fimmu.2019.01973 31481962 PMC6710348

[72] Xie FT Cao JS Zhao J Yu Y Qi F Dai XC. IDO expressing dendritic cells suppress allograft rejection of small bowel transplantation in mice by expansion of Foxp3+ regulatory T cells. Transplant Immunol (2015) 33(2):69–77. doi: 10.1016/j.trim.2015.05.003 26002283

[73] Gandhi R Kumar D Burns E Nadeau M Dake B Laroni A. Activation of the aryl hydrocarbon receptor induces human type 1 regulatory T cell-like and Foxp3(+) regulatory T cells. Nat Immunol (2010) 11(9):846–53. doi: 10.1038/ni.1915 PMC2929008 20676092

[74] Trujillo-Ochoa JL Kazemian M Afzali B. The role of transcription factors in shaping regulatory T cell identity. Nat Rev Immunol (2023) 19. doi: 10.1038/s41577-023-00893-7 PMC10893967 37336954

[75] DiNatale BC Murray IA Schroeder JC Flaveny CA Lahoti TS Laurenzana EM. Kynurenic acid is a potent endogenous aryl hydrocarbon receptor ligand that synergistically induces interleukin-6 in the presence of inflammatory signaling. Toxicological Sci (2010) 115(1):89–97. doi: 10.1093/toxsci/kfq024 PMC2855350 20106948

[76] Gironès N Carbajosa S Guerrero NA Poveda C Chillón-Marinas C Fresno M. Global metabolomic profiling of acute myocarditis caused by trypanosoma cruzi infection. PloS Negl Trop Dis (2014) 8(11):e3337. doi: 10.1371/journal.pntd.0003337 25412247 PMC4239010

[77] Manzella C Singhal M Alrefai WA Saksena S Dudeja PK Gill RK. Serotonin is an endogenous regulator of intestinal CYP1A1 *via* AhR. Sci Rep (2018) 8(1):1–13. doi: 10.1038/s41598-018-24213-5 29666456 PMC5904159

[78] Shinde R McGaha TL. The aryl hydrocarbon receptor: connecting immunity to the microenvironment. Trends Immunol (2018) 39(12):1005–20. doi: 10.1016/j.it.2018.10.010 PMC7182078 30409559

[79] Kaysen GA Dubin JA Müller H-G Mitch WE Rosales LM Levin NW. Relationships among inflammation nutrition and physiologic mechanisms establishing albumin levels in hemodialysis patients. Kidney Int (2002) 61(6):2240–9. doi: 10.1046/j.1523-1755.2002.00076.x 12028466

[80] Badawy AA Guillemin G. The plasma [kynurenine]/[tryptophan] ratio and indoleamine 2, 3-dioxygenase: time for appraisal. Int J Tryptophan Res (2019) 12:1178646919868978. doi: 10.1177/1178646919868978 31488951 PMC6710706

[81] Song PA-O Ramprasath T Wang H Zou MH. Abnormal kynurenine pathway of tryptophan catabolism in cardiovascular diseases. Cell Mol Life Sci (2017) 74(16):2899–2916. doi: 10.1007/s00018-017-2504-2 28314892 PMC5501999

[82] Campbell BM Charych E Lee AW Möller T. Kynurenines in CNS disease: regulation by inflammatory cytokines. Front Neurosci (2014) 8:12. doi: 10.3389/fnins.2014.00012 24567701 PMC3915289

[83] Oxenkrug G Ratner R. N-acetylserotonin and aging-associated cognitive impairment and depression. Aging Disease (2012) 3(4):330. 23185714 PMC3501368

[84] Sun H Gusdon AM Qu S. Effects of melatonin on cardiovascular diseases: progress in the past year. Curr Opin Lipidol (2016) 27(4):408. doi: 10.1097/MOL.0000000000000314 27075419 PMC4947538

[85] Neumann J Hofmann B Gergs U. Production and function of serotonin in cardiac cells. Serotonin-a Chem messenger between all types living Cells (2017) 13:271–305. doi: 10.5772/intechopen.69111

[86] Nikolaus S Schulte B Al-Massad N Thieme F Schulte DM Bethge J. Increased tryptophan metabolism is associated with activity of inflammatory bowel diseases. Gastroenterology (2017) 153(6):1504–16. e2. doi: 10.1053/j.gastro.2017.08.028 28827067

[87] Gulaj E Pawlak K Bien B Pawlak D. Kynurenine and its metabolites in Alzheimer's disease patients. Adv Med Sci (2010) 55(2):204–11. doi: 10.2478/v10039-010-0023-6 20639188

[88] Jacobs KR Castellano-Gonzalez G Guillemin GJ Lovejoy DB. Major developments in the design of inhibitors along the kynurenine pathway. Curr medicinal Chem (2017) 24(23):2471–95. doi: 10.2174/0929867324666170502123114 PMC5748880 28464785

