# Supplementary material for: Association between altered tryptophan metabolism, plasma aryl hydrocarbon receptor agonists, and inflammatory Chagas disease
Source: Front Immunol. 2024 Jan 12;14:1267641. doi: 10.3389/fimmu.2023.1267641 (PMC10811785; doi:10.3389/fimmu.2023.1267641)
Supplement: Supplementary file 1 [file DataSheet_1.pdf]

Supplementary Material

Association between Altered Tryptophan Metabolism, Plasma Aryl Hydrocarbon Receptor Agonists, and Inflammatory Chagas Disease.

Laura Fernanda Ambrosio†[,1,2], Ximena Volpini†[,1,2], Juan Nahuel Quiroz[1,2], María Belén Brugo[1,2], Carolina Paola Knubel[1,2], Melisa Rocío Herrera[1,2], Laura Fozzatti[1,2], Julián Avila Pacheco[5], Clary B. Clish[5], Maisa Takenaka[3], Juan Beloscar[4], Martín Gustavo Theumer[1,2], Francisco Javier Quintana[3,5], Ana Rosa Perez[6] and Claudia Cristina Motrán*[, 1, 2]

1 - Departamento de Bioquímica Clínica, Facultad de Ciencias Químicas, Universidad Nacional de Córdoba

2 - Centro de Investigaciones en Bioquímica Clínica e Inmunología (CIBICI), Consejo Nacional de Investigaciones Científicas y Técnicas (CONICET).

3 – Ann Romney Center for Neurologic Diseases, Brigham and Women's Hospital, Harvard Medical School, Boston, MA, USA.

4 - Servicio de Cardiología, Departamento de Chagas, Hospital Provincial del Centenario y Cátedra de Cardiología, Facultad de Ciencias Médicas, Universidad Nacional de Rosario, Argentina.

5 - Broad Institute of MIT and Harvard, Cambridge, USA.

6 - Instituto de Inmunología Clínica y Experimental de Rosario (IDICER-CONICET-UNR), Argentina; Centro de Investigación y Producción de Reactivos Biológicos (CIPReB), Facultad de Ciencias Médicas, Universidad Nacional de Rosario, Argentina

† These authors share first authorship.

*** Correspondence:**
Motran C.C. , cmotran@unc.edu.ar

Supplementary Figures

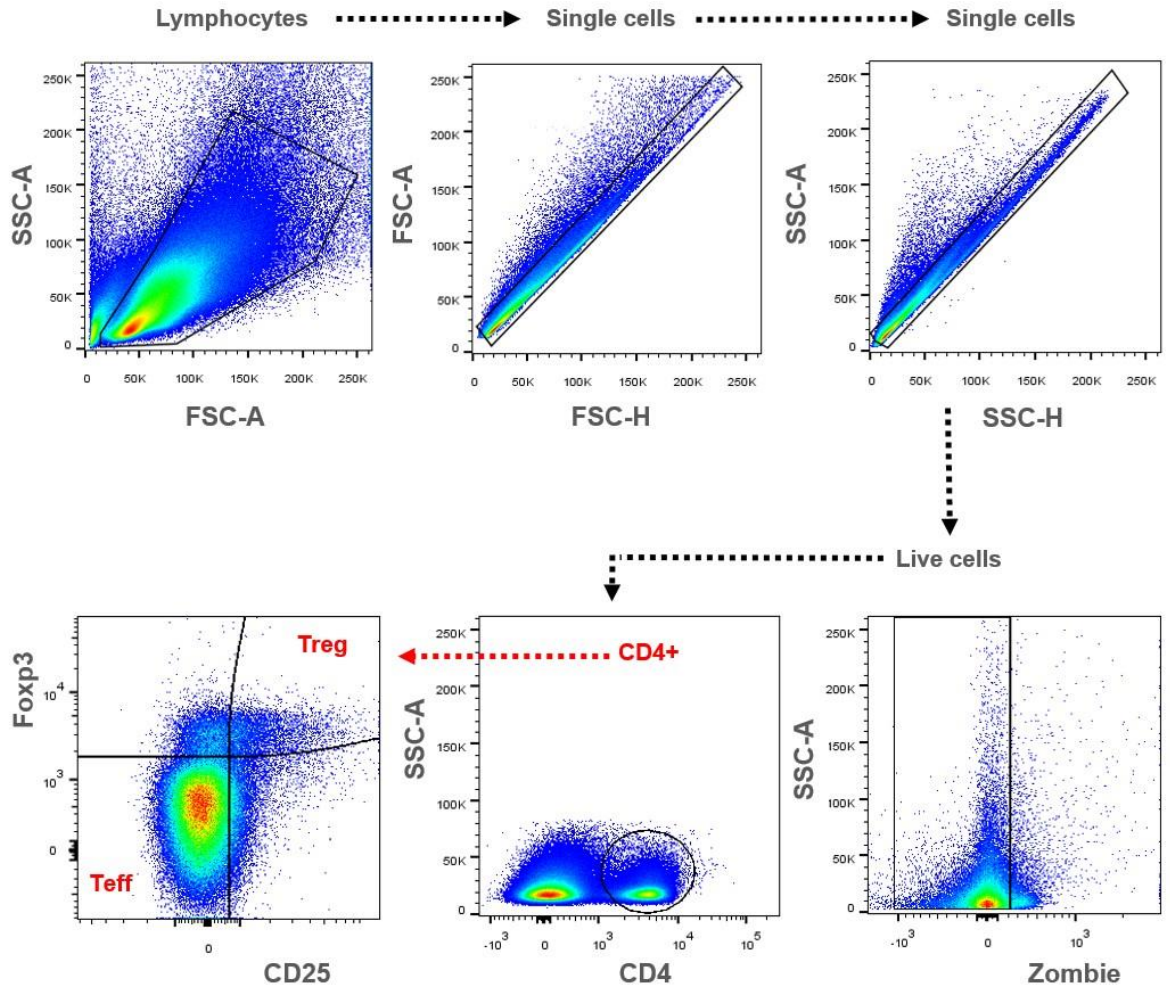

Supplementary Figure 1. Gate strategy of lymphocytes subpopulations.

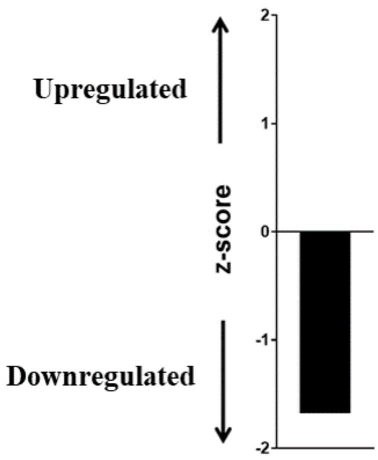

Supplementary Figure 2. Infection of B6-BMDM with *T. cruzi* results in downregulation of AhR pathway-associated genes. B6-BMDM were infected with *T. cruzi* (at a ratio of 1:3, BMDM:Tps) for 24 hours. Following infection, cells were washed and collected for RNA-seq using the High-Throughput 3' Digital Gene Expression (HT-DGE) methodology. Subsequently, the data was analyzed through Ingenuity Pathway Analysis (Qiagen). Comparison of AhR pathway gene expression between *T. cruzi*-infected and non-infected BMDM is shown.

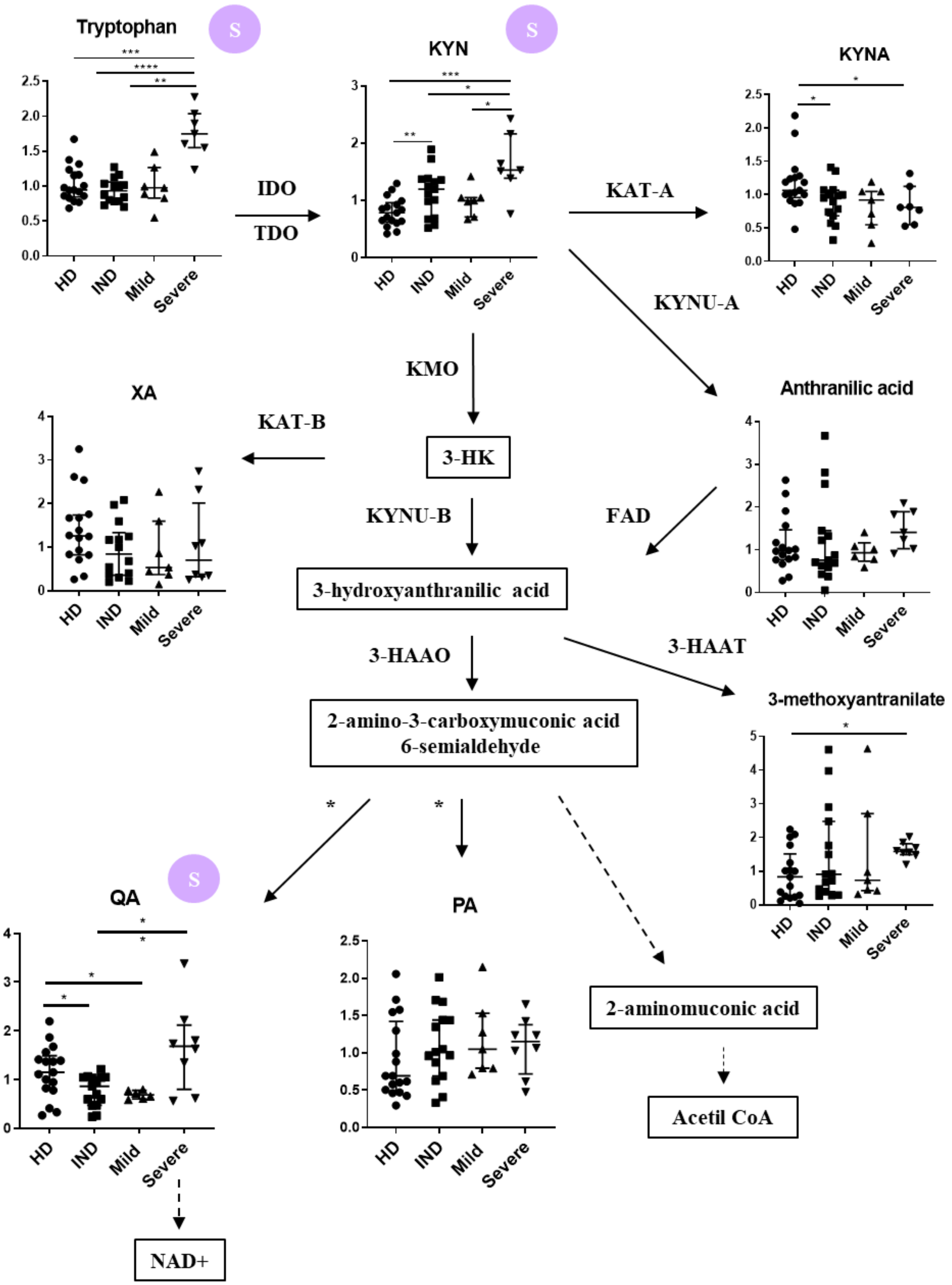

Tryptophan
S
KYN
S
KYNA
IDO
TDO
KAT-A
KYNU-A
KMO
XA
KAT-B
3-HK
Anthranilic acid
KYNU-B
FAD
3-hydroxyanthranilic acid
3-HAAO
3-HAAT
2-amino-3-carboxymuconic acid 6-semialdehyde
3-methoxyantranilate
QA
S
PA
2-aminomuconic acid
Acetil CoA
NAD+
HD
IND
Mild
Severe

Supplementary Figure 3. Kynurenine pathway metabolites detected by LC-MS in plasma from HD and IND, Mild and Severe groups of patients. The median values and inter-quartile range of the normalized ion abundance in plasma samples are shown. IDO: indoleamine 2,3-dioxigenase; TDO: tryptophan 2,3-dioxygenase, KAT: kynurenine aminotransferase; KYNU: kynureninase; KMO: kynurenine 3-monooxygenase; 3-HAAO: 3-hydroxyanthranilate 3,4-dioxygenase; FAD: anthranilate 3-monooxygenase; 3-HAAT: 3-hydroxyanthranilate O-methyltransferase; NAD: nicotinamide adenine dinucleotide. Statistically significant differences were calculated using the Mann Whitney U-test. (*) $p \leq 0.05$; (**) $p \leq 0.01$; (***) $p \leq 0.001$; and (****) $p \leq 0.0001$. S: indicates the metabolites that contribute with Severe group PCA segregation.

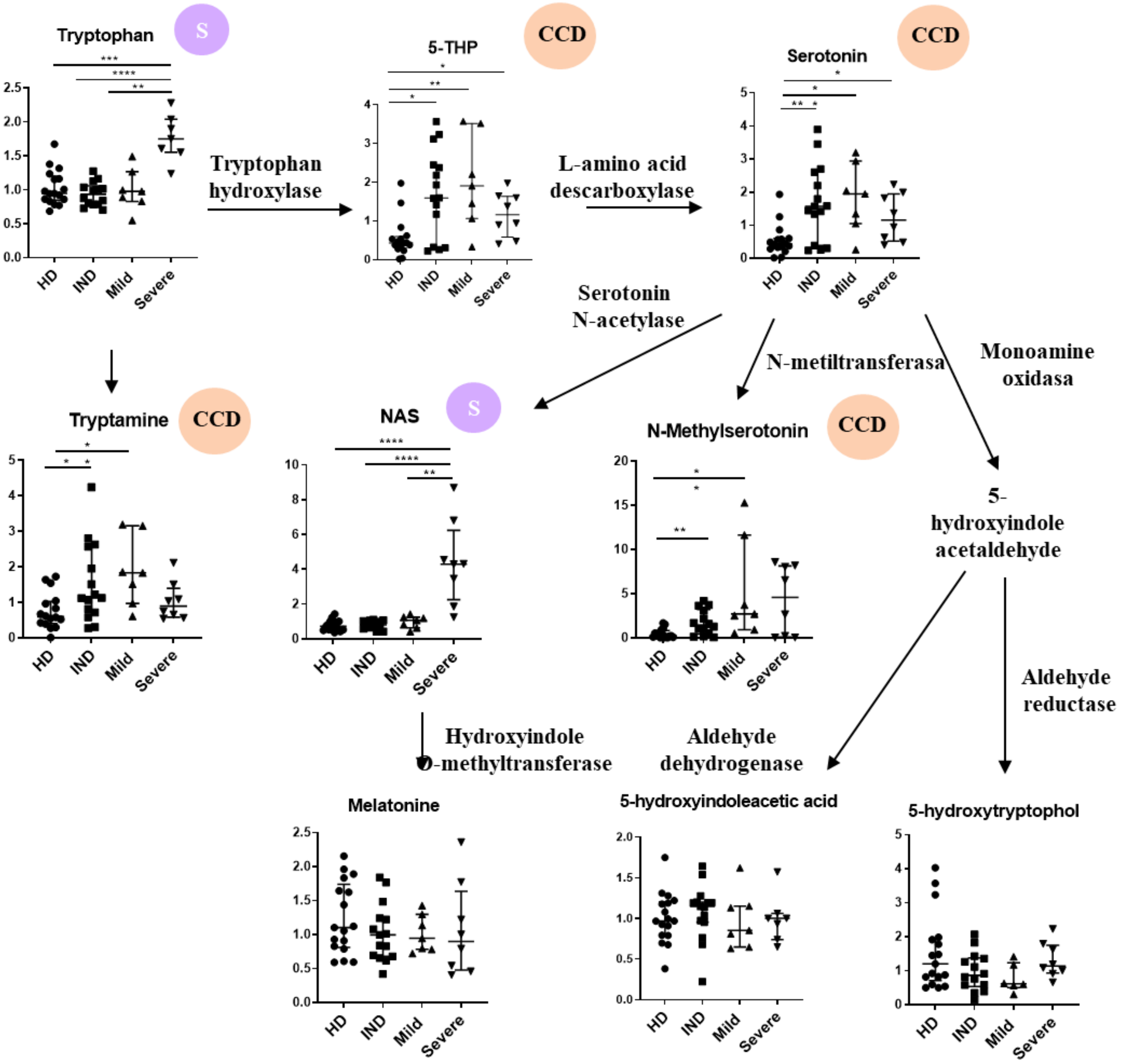

Supplementary Figure 4. Serotonin and tryptamine pathways metabolites detected by LC-MS in plasma from HD and IND, Mild and Severe groups of patients. The median values and inter-quartile range of the normalized ion abundance in plasma samples are shown. Statistically significant differences were calculated using the Mann Whitney U-test. (*) $p \leq 0.05$; (**) $p \leq 0.01$; and (***) $p \leq 0.001$. S: indicates the metabolites that contribute with Severe group PCA segregation CCD: indicates the metabolites that contribute with CCD patients PCA segregation.

HD

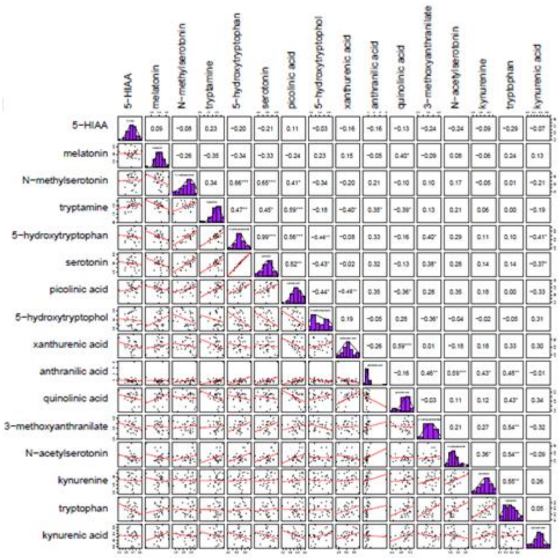

Indeterminate

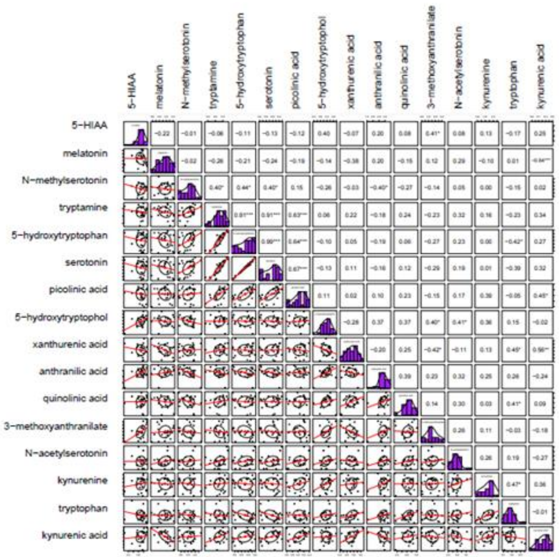

Mild

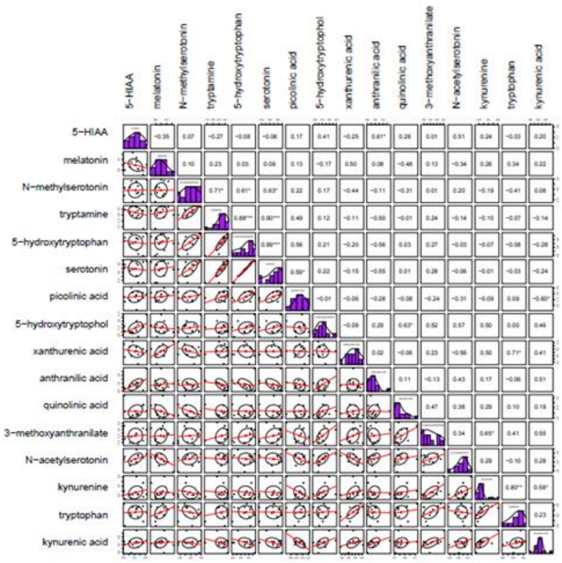

Severe

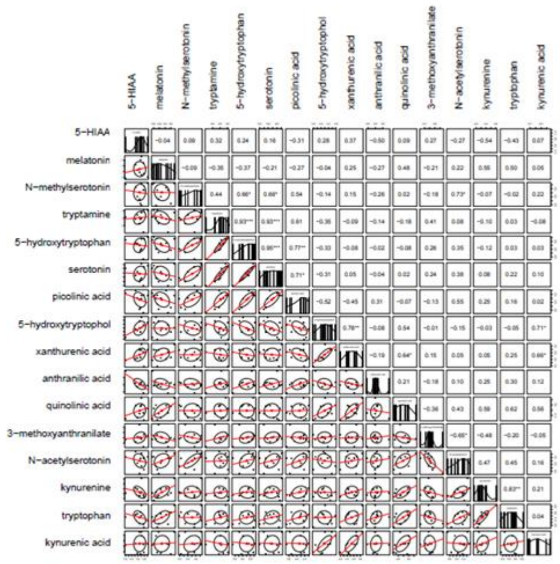

Supplementary Figure 5. Pairwise-multiple correlation, obtained using R, across the relative ion abundance of Trp metabolites in the plasma samples from HD and CCD patients. CCD patients were classified as IND, Mild, and severe. The distribution of each metabolite is displayed as a histogram. The scatter chart with trendline (left) and Spearman's rank correlation coefficient (right) for each comparison are depicted. The strength of correlation was defined as weak (0.00-0.39), moderate (0.40-0.59) or strong (0.60-1.00). Each correlation was considering as statistically significant with a p value less than 0.05. (*) p <0.05; (**)p <0.01 (***); and p <0.001.

Table 1

Figure	Cohorte	Females/Male	Age
1A	Control (17)	13/4	46.52 (39-53)
	INF (29)	20/9	49.48 (41-53)
1B	Control (25)	16/9	42.15 (37-50)
	IND (18)	12/6	45.61 (40-53)
	CCC (18)	12/6	46.5 (41-55)
1C	Control (17)	12/5	43.37 (41-54)
	IND (15)	10/5	49.61 (40-57)
	Mild (7)	6/1	50.14 (41-55)
	Severe (7)	5/2	48.85 (42-53)

Table 1. Clinical and demographic characteristics of the cohort. Each group is denoted by its respective name as displayed in the corresponding figure, with the sample size (n) provided in brackets. Sex: The numbers represent the absolute number of female individuals (biological sex) from left to right, followed by the percentage of females in the group. Age: The average age is indicated, with the 25% and 75% percentiles provided in parentheses. For males, the overall mean age was 46 years, and 41 for females. The prevalent manifestation of Chagas disease was the indeterminate form (53%), followed by the cardiac form (45%). Mild cardiac patients showing any of the following alterations by ECG: incomplete right bundle branch block (47%) or complete (23%), first degree of atrioventricular block (32%) or non-life-threatening arrhythmias (76%). Patients with severe myocarditis, presenting

major ECG pathological tracings, that is, complex ventricular arrhythmia (53%) or complete atrioventricular block (68%) and/or congestive heart failure (73%). Additionally, systemic arterial hypertension emerged as a common comorbidity, affecting 53% of the patients.
